# A Ubiquitin‐Dependent Switch on MEF2D Senses Pro‐Metastatic Niche Signals to Facilitate Intrahepatic Metastasis of Liver Cancer

**DOI:** 10.1002/advs.202305550

**Published:** 2023-10-12

**Authors:** Junyu Xiang, Ni Zhang, Aibei Du, Jinyang Li, Mengyun Luo, Yuzhu Wang, Meng Liu, Luming Yang, Xianfeng Li, Lin Wang, Qin Liu, Dongfeng Chen, Tao Wang, Xiu‐wu Bian, Zhong‐yi Qin, Li Su, Liangzhi Wen, Bin Wang

**Affiliations:** ^1^ Department of Gastroenterology Chongqing Key Laboratory of Digestive Malignancies Daping Hospital Army Medical University (Third Military Medical University) Chongqing 400042 China; ^2^ Institute of Pathology and Southwest Cancer Center and Key Laboratory of Tumor Immunopathology of Ministry of Education of China Southwest Hospital Army Medical University (Third Military Medical University) Chongqing 400038 China; ^3^ Department of Oncology and Hematology Chongqing Hospital of Traditional Chinese Medicine Chongqing 400030 China; ^4^ Jinfeng Laboratory Chongqing 401329 China

**Keywords:** disseminated cancer cells, integrin, MEF2D, neutrophil extracellular traps, pro‐metastatic niche

## Abstract

Effective treatment for metastasis, a leading cause of cancer‐associated death, is still lacking. To seed on a distal organ, disseminated cancer cells (DCCs) must adapt to the local tissue microenvironment. However, it remains elusive how DCCs respond the pro‐metastatic niche signals. Here, systemic motif‐enrichment identified myocyte enhancer factor 2D (MEF2D) as a critical sensor of niche signals to regulate DCCs adhesion and colonization, leading to intrahepatic metastasis and recurrence of liver cancer. In this context, MEF2D transactivates *Itgb1* (coding β1‐integrin) and *Itgb4* (coding β4‐integrin) to execute temporally unique functions, where ITGB1 recognizes extracellular matrix for early seeding, and ITGB4 acts as a novel sensor of neutrophil extracellular traps‐DNA (NETs‐DNA) for subsequent chemotaxis and colonization. In turn, an integrin‐FAK circuit promotes a phosphorylation‐dependent USP14‐orchastrated deubiquitination switch to stabilize MEF2D via circumventing degradation by the E3‐ubiquitin‐ligase MDM2. Clinically, the USP14(pS432)‐MEF2D‐ITGB1/4 feedback loop is often hyper‐active and indicative of inferior outcomes in human malignancies, while its blockade abrogated intrahepatic metastasis of DCCs. Together, DCCs exploit a deubiquitination‐dependent switch on MEF2D to integrate niche signals in the liver mesenchyme, thereby amplifying the pro‐metastatic integrin‐FAK signaling. Disruption of this feedback loop is clinically applicable with fast‐track potential to block microenvironmental cues driving metastasis.

## Graphical abstract

In response to pro‐metastatic niche signals in liver, disseminated cancer cells (DCCs) exploit a deubiquitination‐dependent switch on MEF2D to up‐regulate ITGB1 and ITGB4, where ITGB4 acts as a novel receptor of NETs‐DNA. Combined blockade of ITGB1 and ITGB4 may be a promising approach to inhibit intrahepatic colonization of DCCs, thereby preventing cancer metastasis and recurrence.

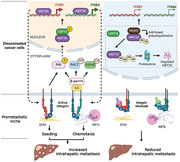

## 1 Introduction

Cancer metastasis is an inefficient process consisting of primary tumor infiltration, intravasation and survival of disseminated cancer cells (DCCs) in the vasculature, extravasation, seeding and colonization of distal tissues. Along this multi‐step cascade, seeding and colonization of DCCs in an unfamiliar microenvironment is the most challenging and rate‐limiting step.^[^ ^1^ ^]^ To successfully colonize the recipient tissue, DCCs must integrate various supportive signals in the pro‐metastatic niche. Whereas there is growing interest in the structural and functional reprogramming of the niche components,^[^ ^2^, ^3^, ^4^ ^]^ it remains poorly understood how DCCs sense various niche signals to allow efficient seeding and colonization.

Upon reaching distal tissue sites, DCCs interact with components of the pro‐metastatic niche for adhesion and survival signals.^[^ ^5^ ^]^ The acellular extracellular matrix (ECM) is recognized by receptor integrins clustering on DCCs to promote physical anchorage through activating focal adhesion kinase (FAK) and SRC signaling.^[^ ^6^ ^]^ Various cellular infiltrates in the developing metastatic microenvironment, including immune cells, further foster chemotaxis, growth advantage, and immunoevasion properties to DCCs.^[^ ^7^ ^]^ Notably, neutrophils enriched in the liver and lung, the most frequent sites of metastatic cancer, extrude a web of chromatin DNA filaments called neutrophil extracellular traps (NETs) to constitute a unique niche component.^[^ ^8^ ^]^ As expression of CCDC25, a NETs receptor, differs greatly in various cancer types, it is unclear whether there are other sensors for NETs signals. Nonetheless, disruption of pro‐metastatic niche signals represents an important approach to eliminate DCCs.

Pro‐metastatic niche signals converge on a specific set of transcription factors (TFs) to dictate cell fate of DCCs. To this end, TFs are typically enriched on several binding motifs in the regulatory regions of niche signaling signature genes. For example, matrix stiffness through integrin‐ILK signaling activates the YAP/TAZ transcriptional complex to regulate cell growth.^[^ ^9^ ^]^ However, few TFs have been identified for DCCs that integrate pro‐metastatic niche signals. It is also largely unknown how such TFs facilitate metastatic colonization and how they are dynamically regulated by various niche signals.

As one of the most lethal cancers, hepatocellular carcinoma (HCC) is incline to disseminate intrahepatically, leading to a high rate of post‐surgery recurrence.^[^ ^10^, ^11^ ^]^ It is of paramount importance to elucidate how the disseminated HCC cells co‐opt the pro‐metastatic niche signals for intrahepatic spreading. Here, we identify myocyte enhancer factor 2D (MEF2D) as an essential TF that transactivates *Itgb1* and *Itgb4* to sense ECM and NETs‐DNA components for early seeding and subsequent colonization, respectively. Unlike a role of MEF2D in enhancing growth and immune escape of HCC cells,^[^ ^12^, ^13^, ^14^, ^15^ ^]^ and epithelial‐mesenchymal transition (EMT) of colorectal cancer cells,^[^ ^16^ ^]^ MEF2D promotes the spreading of HCC cells independent of regulating the EMT. Moreover, niche signals induce a USP14‐governed ubiquitin switch to stabilize MEF2D and constitute a feedback loop to amplify the MEF2D‐integrin axis. Thus, our findings provide a rationale for combined blockade of ITGB1 and ITGB4, with clinically available antibodies, as a promising therapeutic strategy for management of HCC intrahepatic metastasis.

## 2 Results

### 2.1 Identification of MEF2D as a Central Coordinator of the Pro‐Metastatic Niche Signals in DCCs to Promote Cell Adhesion, Intrahepatic Dissemination, and Disease Recurrence of HCC

**Figure 1:**
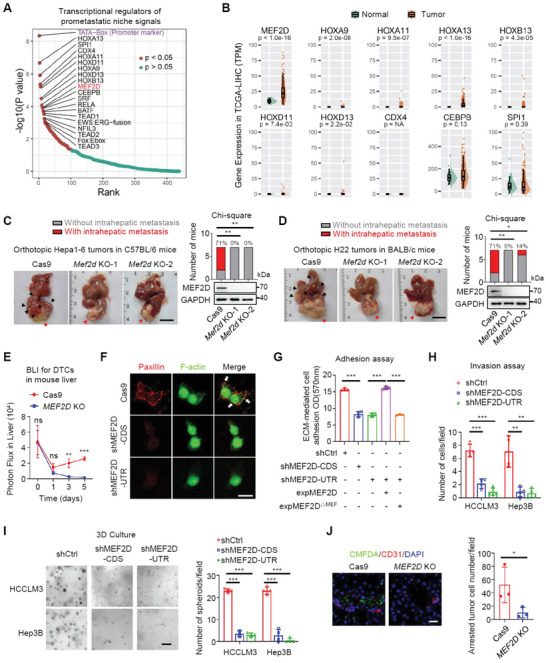
An unbiased screen identifies MEF2D as a core transcription factor integrating pro‐metastatic niche signals to promote intrahepatic metastasis of disseminated HCC cells. A) The top 20 TF motifs enriched at the promoter of signature genes among various pro‐metastatic niche signaling pathways. B) Differential expression of the top 10 enriched TFs in (A) between normal and tumor samples from TCGA‐LIHC project. C,D) Intrahepatic metastases generated by the orthotopically inoculated *Mef2d*‐depleted and control Hepa1‐6 cells in livers from syngeneic C57BL/6 mice (C), and H22 cells in livers from syngeneic BALB/c mice (D) (n = 7). Red arrow indicates primary tumor, while black arrow indicates metastases. Scale bars, 1 cm. The knockout efficiency of *Mef2d* in each line were analyzed by immunoblotting as shown in each panel. E) BLI at 0–5 days after splenic injection of *Mef2d*‐depleted and control HCCLM3 cells (n = 5‐6). F) Redistribution of focal adhesions and cytoskeletal remodelling in sh*Mef2d* and control Hep3B cells on the ECM were examined by double immunostaining for paxillin (red) and F‐actin (green). Yellow staining in merged images represents colocalization of paxillin with actin filaments. White arrows indicate focal adhesions. sh*Mef2d*‐CDS or sh*Mef2d*‐UTR are shRNAs targeting the CDS or UTR region of *Mef2d* mRNA, respectively. Scale bars, 20 µm. G) Crystal violet staining‐based quantification of cell adhesion assay using shMEF2D or control Hep3B cells, or *Mef2d*‐depleted cells transfected with wild‐type or a mutant MEF2D lacking MEF domain (MEF2D^△MEF^). H) Quantitation of invaded cells in an ECM‐coated transwell assay. I) 3D growth of wild‐type or *MEF2D*‐depleted cells in ECM. Scale bar, 200 µm. J) Intrahepatic seeding of wild‐type or *Mef2d*‐depleted HCCLM3 cells. CMFDA (green)‐labelled cells were injected into the spleen of mice for 48 h. Liver slices were stained for immunofluorescence with CD31 (red) (n = 3). Numbers of seeded cells were quantified from 10 random fields of each liver. Scale bars, 20 µm. All immunoblots are representative experiments of three independent replicates. For all panels, * *p* < 0.05, ** *p* < 0.01, *** *p* < 0.001, and ns, no significance.

To unbiasedly identify transcriptional regulators of DCCs, we collected 102 gene signatures governed by various signaling pathways of the pro‐metastatic niche in the liver,^[^ ^7^, ^17^ ^]^ including cell‐ECM interactions and focal adhesion (Table S1, Supporting Information). Motif enrichment analysis identified several TFs (**Figure** **1**A; Figure S1A, Supporting Information), such as those from the HOX and Hippo‐TEAD families with known roles in tumor metastasis.^[^ ^9^, ^18^ ^]^ However, among the top 10 enriched TFs, only MEF2D was highly expressed in HCC tissues, while the expression levels of the other identified TFs were extremely low (HOX family and CDX4) or comparable to normal tissues (CEBPB and SPI1) (Figure 1B). Expression of MEF2D, but not CEBPB, was positively correlated with the many pro‐metastatic signatures, especially the integrin‐FAK pathway (Figure S1B, Supporting Information). The MEF2D signature was also higher in the transcriptome of intrahepatic metastases than that of the primary HCC lesions (Figure S1C, Supporting Information), indicating that MEF2D may be a potential integrator of pro‐metastatic niche signals.

Consistently, knocking out (KO) *MEF2D* in murine HCC cells significantly inhibited intrahepatic metastasis of orthotopic tumors in syngeneic immunocompetent mice (Figure 1C,D). A similar phenotype was also observed when using highly metastatic human HCC cells for both intrahepatic and spleen‐to‐liver metastasis assays (Figure S1E–G, Supporting Information). Gene set enrichment analysis (GSEA) revealed an enrichment of a MEF2D‐dependent intrahepatic metastasis gene signature^[^ ^19^ ^]^ (Figure S1H, Supporting Information). These results suggest that MEF2D plays an essential role in sustaining intrahepatic metastasis of DCCs.

Monitoring the temporal course of metastasis revealed that depletion of *Mef2D* led to impaired intrahepatic seeding as early as 3‐ and 5‐days post‐inoculation (Figure 1E). Thus, we first examined whether MEF2D promotes epithelial‐mesenchymal transition (EMT), an initiating event of the metastatic cascade. Unexpectedly, MEF2D was neither associated with the EMT phenotypes during liver tumorigenesis in *Alb‐cre^+^;Mef2d^fl/fl^* mice,^[^ ^14^ ^]^ nor the EMT signature in human HCC tissues (Figure S1I–M, Supporting Information). Thus, MEF2D promotes early metastatic seeding of DCCs without affecting the EMT process.

To characterize a precise role of MEF2D in the metastasis cascade, depleting *Mef2d* suppressed focal adhesion formation, leading to impaired adhesion to the ECM. Restoring the wild‐type MEF2D, but not the dominant‐negative MEF2D^△MEF^ truncation, rescued cellular adhesion (Figure 1F,G; Figure S1N, O, Supporting Information). Conversely, ectopic expression of MEF2D in cells with low endogenous levels of MEF2D increased their focal adhesions and attachment on ECM (Figure S1D,P,Q, Supporting Information). Consequently, *Mef2d*‐depleted cells were less able to invade and survive in ECM, resulting in impaired seeding in the liver mesenchyme (Figure 1H–J). On the other hand, ectopic expression of MEF2D promoted anchorage‐independent growth in ECM (Figure S1R, Supporting Information). Collectively, MEF2D facilitates the adhesion of DCCs to ECM components of the pro‐metastatic niche, thereby sustaining seeding for subsequent intrahepatic colonization.

Intrahepatic metastasis is causally linked to recurrence.^[^ ^10^, ^11^ ^]^ Consistently, higher levels of MEF2D or MEF2B, but not other members of the MEF2 family, correlated to accelerated recurrence, while only high expression of MEF2D was linked to shorter survival of patients (Figure S2A–C, Supporting Information). Immunostaining of other HCC cohort samples^[^ ^12^ ^]^ confirmed that high level of MEF2D protein was associated with earlier recurrence after surgery (Figure S2D, Supporting Information). Together, MEF2D may be an essential coordinator of pro‐metastatic niche signaling to enhance intrahepatic metastasis and post‐surgery recurrence.

### 2.2 ITGB1 and ITGB4, Two Transcriptional Targets of MEF2D, Execute Temporally Distinct Functions to Sustain Early DCCs Seeding and Later Colonization, Respectively

**Figure 2:**
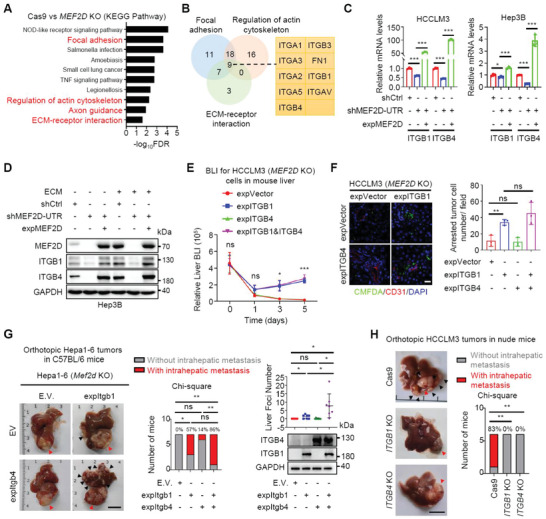
ITGB1 and ITGB4 play distinct regulatory roles at early seeding and late colonization stages of MEF2D‐driven dissemination of DCCs in the liver. A) Kyoto Encyclopedia of Genes and Genomes (KEGG) enrichment analysis of downregulated pathways in *Mef2d*‐depleted cells compared to control cells. B) Venn diagram of differentially expressed genes (DEGs) in (A) that were associated with the pathways of focal adhesion, regulation of actin cytoskeleton, and ECM‐receptor interaction. C) qRT‐PCR analysis of *Itgb1* and *Itgb4* expression in control, *Mef2d*‐depleted, and sh*Mef2d* cells reconstituted with *Mef2d* (HCCLM3 or Hep3B). D) Immunoblot analysis of ITGB1 and ITGB4 levels in sh*Mef2d* and control Hep3B cells, and sh*Mef2d* cells reconstituted with *Mef2d*. Culture plates were coated with or without ECM. Panel is representative of three independent replicates. E) BLI at 0–5 days after splenic injection of *Mef2d*‐depleted HCCLM3 cells that were reconstituted with either *Itgb1* or *Itgb4*, or both (n = 5‐6). F) Intrahepatic seeding of the indicated HCCLM3 cells as visualized by immunofluorescence staining. CMFDA (green)‐labelled cells were injected into the spleen and liver with seeding monitored 48 h later. Scale bars, 20 µm. G) Intrahepatic metastases of the orthotopically injected *Mef2d*‐depleted Hepa1‐6 cells that were ectopically expressing either *Itgb1* or *Itgb4*, or both (n = 7). Red arrow, primary tumor; black arrow, metastases. Scale bars, 1 cm. The expression efficiency of *Itgb1* and *Itgb4* were analyzed by immunoblot. H) Intrahepatic metastases of the orthotopically injected *Itgb1*‐ or *Itgb4*‐depleted HCCLM3 cells in nude mice (n = 6). Red arrow, primary tumor; black arrow, metastases. Scale bars, 1 cm. All immunoblots are representative experiments of three independent replicates. For all panels, * *p* < 0.05, ** *p* < 0.01; *** *p* < 0.001, and ns, no significance.

Analysis of transcriptomic profiles of *Mef2d*‐KO cells identified reduced expression of integrin family members that regulate focal adhesion and ECM‐receptor interaction (**Figure** **2**A,B). Consistently, MEF2D positively correlated with these highly expressed integrins in HCC and other cancer types (Figure S3A–G, Supporting Information). Among them, only ITGB1 and ITGB4 were constantly up‐regulated in a MEF2D dependent manner (Figure 2C,D; Figure S3C–E, Supporting Information). As a TF, MEF2D occupied binding sites in the promoter and enhancer site 2 of *Itgb1*, and the enhancer site 2 of *Itgb4* to regulate their transcriptional activities (Figure S4A–E, Supporting Information). Moreover, modulating the expression of MEF2D regulated multiple activating (H3K18ac, H3K27ac, H4K8ac, and H4K16ac, and H3K4me3) and repressive (H3K9m2) histone markers (Figure S4F,G, Supporting Information). Thus, MEF2D enrichment elicits extensive epigenetic reprogramming to activate transcription of *Itgb1* and *Itgb4*.

Re‐introducing ITGB1, but not ITGB4, in *Mef2d*‐depleted cells was sufficient to rescue both early seeding (Figure 2E,F) and later metastatic dissemination (Figure 2G,H) in the liver. Consistently, restoring the expression of ITGB1, but not ITGB4, phenocopied the effect of MEF2D on focal adhesion formation, invasion, and three‐dimensional (3D) growth of DCCs in the ECM (Figure S5A‐I, Supporting Information). Due to integrin loss, *Mef2d*‐KO cells displayed attenuated activities of FAK signaling and SRC signaling in the presence of ECM components, which was rescued by ITGB1, but not ITGB4 (Figure S5J, Supporting Information). Moreover, FAK activity was essential for MEF2D‐ and ITGB1‐driven cell adhesion, invasion, and 3D growth in the ECM (Figure S5K–R, Supporting Information). In human liver cancers, MEF2D expression was correlated with FAK signatures (Figure S5S, Supporting Information), while inhibition of either MEF2D or FAK downregulated a panel of genes essential for cell adhesion and metastasis^[^ ^20^ ^]^ (Figure S5T, Supporting Information). These data demonstrate that ITGB1, but not ITGB4, is pivotal for DCCs adhesion to the ECM and activation of FAK signaling, thereby enabling seeding in the pro‐metastatic niche.

Interestingly, although re‐introducing ITGB4 alone could not support initial DCCs seeding (Figure 2E,F), restoration of ITGB4 in cells expressing ITGB1 increased visible metastatic nodules with a significantly higher tumor burden, as compared to cells expressing ITGB1 alone. These results were consistently observed both in immunocompetent mice bearing orthotopic HCC tumors and in a spleen‐to‐liver metastatic model in nude mice (Figure 2G; Figure S5U,V, Supporting Information). Moreover, in HCCLM3 cells that expressed high levels of endogenous MEF2D, depleting ITGB4 significantly suppressed metastasis (Figure 2H). Hence, unlike the critical role of ITGB1 during the full metastatic processes, ITGB4 appears to be uniquely necessary for later colonization stages of DCCs in an ITGB1‐dependent manner in vivo.

### 2.3 ITGB4, a Canonical Extracellular Matrix Receptor, Acts as a Novel Receptor for Neutrophil Extracellular Traps in the Pro‐Metastatic Niche to Sustain Colonization of DCCs at Later Metastasis Stages

**Figure 3:**
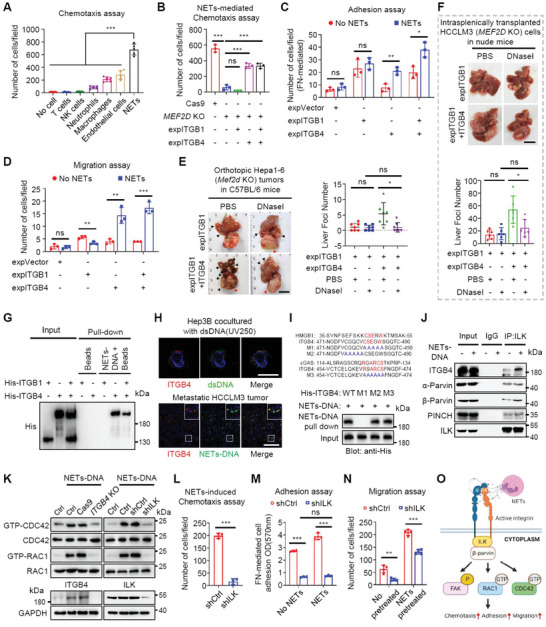
ITGB4, a transcriptional target of MEF2D, recognizes NETs‐DNA to sustain metastatic colonization. A) Chemotaxis assays with Hepa1‐6 cells in the upper chambers and culture media and indicated murine cell components of the pro‐metastatic niche in the lower chambers of transwell assays. Tumor cells:other cells = 1:5. B) Chemotaxis assay with *Mef2d*‐depleted Hep3B cells reconstituted with either *Itgb1*, or *Itgb4*, or both, in the in upper chambers and NETs added to the culture media in the lower chambers. Tumor cells:NETs = 1:5. C,D) Adhesion (C) and migration (D) assays for various *Mef2d*‐depleted Hep3B cells stimulated with or without 5 µg mL^−1^ NETs. E) Intrahepatic metastases of the orthotopically injected *Mef2d*‐depleted Hepa1‐6 cells that were reconstituted with either *Itgb1*, or both *Itgb1* and *Itgb4* in C57BL/6 mice and subsequently treated with DNase I (5 mg k^−1^ g) (n = 7). Red arrow, primary tumor; black arrow, metastases; Scale bars, 1 cm. F) Representative images of liver metastases of *Mef2d*‐depleted HCCLM3 cells reconstituted with either *Itgb1*, or both *Itgb1* and *Itgb4*. The cells were injected into spleens of nude mice and subsequently treated with DNase I. The numbers of liver metastases were counted (n = 6). Scale bar, 1 cm. G) Purified His‐tagged ITGB1 and/or His‐tagged ITGB4 proteins were incubated in the presence or absence of biotinylated NETs‐DNA. The bound proteins were immunoprecipitated with streptavidin microbeads and immunoblotted with an anti‐His antibody. H) Representative immunofluorescence images staining for ITGB4 and dsDNA or NETs‐DNA in Hep3B cocultured with dsDNA (top) or in metastatic HCCLM3 tumors (bottom). Yellow staining in merged images represents the areas of NETs‐DNA colocalization with ITGB4. Scale bars, 25 µm (top), 100 µm (bottom). I) Sequence alignment of the extracellular domain of ITGB4 with the DNA‐binding domains of two classical DNA sensors HMGB1 and cGAS. The interaction between His‐tagged full‐length ITGB4 (WT), the AA_481‐485_ mutant (M1), the AA_476‐480_ mutant (M2), or the AA_464‐469_ mutant (M3) with NETs‐DNA. NETs‐DNA was precipitated and immunoblotted using anti‐His antibody. J) Immunoblotting of ILK, β4‐integrin, α‐Parvin, β‐Parvin and PINCH in lysates (input) or anti‐ILK immunoprecipitates from Hep3B cells treated with or without NETs. IgG serves as an isotype control for IP assay. K) Hep3B cells were depleted of either ITGB4 or ILK, and then stimulated with or without NETs. GTP‐bound or total RAC1 and CDC42 levels were examined in the cell lysates. Ctrl, wild‐type cells without transfection. L) Chemotaxis assay for *ILK*‐depleted or control Hep3B cells in (K) in the upper chambers and NETs added to the culture media in the lower chambers of transwell assays. Tumor cells:NETs = 1:5. M,N) Adhesion (M) and migration (N) assays using *ILK*‐depleted or control Hep3B cells in (K) that were stimulated with or without 5 µg mL^−1^ NETs. O) A schematic model of ITGB4‐mediated recognition of NETs‐DNA and its downstream signalling pathways. All immunoprecipitation and immunoblots are representative experiments of three independent replicates. For all panels * *p* < 0.05, ** *p* < 0.01, *** *p* < 0.001, and ns, no significance.

Since ITGB4 did not mediate DCCs‐ECM interaction (Figure 2E,F; Figure S5A–D,J, Supporting Information), we thought it might function through recognizing other components of the pro‐metastatic niche at a later DCCs colonization stage. First, we examined various cellular components at both early seeding stages (4 days) and later colonization stages (30 days). Neutrophils, their derivative NETs, and CD3^+^ T cells, but not other cell types, were significantly increased in both metastatic tumors and their adjacent tissues at later stages (Figure S6A, Supporting Information), as is the case in breast cancer metastases to the liver.^[^ ^8^ ^]^ Functionally, NETs significantly enhanced chemotaxis of HCC cells (**Figure** **3**A; Figure S6B, Supporting Information). Moreover, NETs‐DNA substantially promoted adhesion of DCCs to ECM components and subsequent chemotaxis (Figure S6C, D, Supporting Information). Thus, NETs enriched in later stage metastasis and adjacent tissues may be an important chemoattractant to sustain colonization of DCCs.

We next investigated whether the MEF2D‐ITGB1/4 axis was linked to NETs in regulating metastasis. Depleting *Mef2d* abolished NETs‐mediated chemotaxis and this phenotype was primarily mediated by ITGB4 but not ITGB1 (Figure 3B; Figure S6E,F, Supporting Information). Consistently, ITGB4 but not ITGB1 was both necessary and sufficient for NETs‐induced cell adhesion, migration, and growth (Figure 3C,D; Figure S6G–I, Supporting Information). Treating tumor‐bearing mice with DNase I, to degrade NETs‐DNA,^[^ ^8^ ^]^ specifically blocked ITGB4 but not ITGB1‐driven metastatic colonization (Figure 3E,F; Figure S6J,K, Supporting Information). Thus, following initial intrahepatic seeding, DCC colonization at later stages may be sustained by NETs in the pro‐metastatic niche in an ITGB4‐dependent manner.

We were interested to further define how ITGB4 could transduce NETs signals. An in vitro DNA‐binding assay revealed that ITGB4, but not ITGB1, interacted with NETs‐DNA (Figure 3G,H) and moreover the interaction was specifically with DNA, but not DNA‐associated proteins (Figure S7A, Supporting Information). Furthermore, ITGB4 display stronger interactions with 8‐OHdG‐enriched DNA, a process that was visualized in vitro and in vivo, and abrogated by depleting *ITGB4* (Figure 3H; Figure S7B‐D, Supporting Information). Therefore, it appears that ITGB4, a canonical ECM ligand receptor, acts as a novel receptor for NETs‐DNA.

To dissect the mechanisms of ITGB4‐NETs‐DNA interaction, two potential amino acid motifs (AA_481‐485_ and AA_464‐469_) were identified in the extracellular domain of ITGB4 (Figure 3I), resembling DNA‐binding domain of HMGB1 and cGAS, respectively. Mutation of AA_481‐485_, but not other motifs_,_ abrogated ITGB4‐NETs‐DNA interaction (Figure 3I), suggesting that this motif mediates their direct interaction.

We next dissected how ITGB4 could transduce NETs‐DNA‐induced intracellular signaling. NETs‐DNA enhanced endogenous binding of ITGB4 to integrin‐linked kinase (ILK) (Figure 3J), a putative mediator of NETs‐driven cancer metastasis,^[^ ^8^, ^21^ ^]^ leading to recruitment of β‐parvin, but not α‐parvin or PINCH, to mobilize the small GTPases RAC1 and CDC42 (Figure 3J,K). In addition, NETs‐DNA induced phosphorylation of FAK but not SRC, and treatment with DNaseI, or depletion of either *ITGB4* or *ILK*, blocked this effect (Figure S7E–G, Supporting Information). As such, NETs‐DNA, via ITGB4, induced chemotaxis, cell adhesion, and migration of DCCs via ILK signaling (Figure 3L–N).

Interestingly, these processes were not disrupted by deleting *CCDC25* (Figure S7I, Supporting Information), a known NETs‐DNA sensor in breast cancer.^[^ ^8^ ^]^ *CCDC25* depletion only showed a modest effect on NETs‐mediated HCC cell chemotaxis, likely due to its lower expression in HCC than in breast cancer (Figure S7J,K, Supporting Information). Thus, ITGB4, a novel sensor of extracellular NETs‐DNA, recruits the ILK‐β‐parvin complex independently of CCDC25 to activate FAK and RAC1/CDC42 signaling, thereby sustaining MEF2D‐driven intrahepatic colonization of DCCs at later metastatic stages (Figure 3O).

### 2.4 Pro‐Metastatic Niche Signals Stabilize MEF2D via Inhibiting its Poly‐Ubiquitination and Degradation through an Integrin‐FAK Positive Feedback Loop

**Figure 4:**
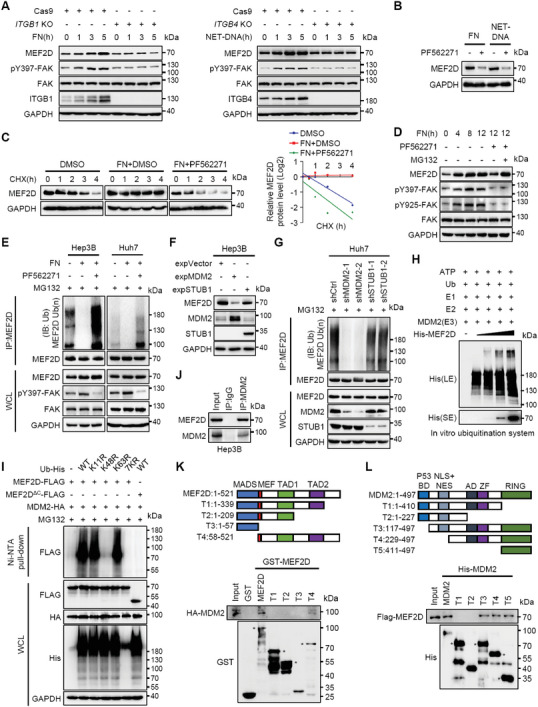
Signals from the pro‐metastatic niche inhibit MDM2‐mediated ubiquitination and proteasomal degradation of MEF2D via an integrin‐FAK feedback loop. A) Immunoblot analysis of pY397‐FAK and MEF2D protein levels in *ITGB1*‐ or *ITGB4*‐depleted Hep3B cells that were treated with FN (bottom coated, 10 µg ml^−1^) or NETs‐DNA (5 µg ml^−1^), respectively, for the indicated times. B) Immunoblot analysis of MEF2D protein levels in Hep3B cells incubated with FN or NETs‐DNA in the presence or absence of the FAK inhibitor PF562271 (10 µM) for 12 h. C) Cycloheximide chase assay to measure the stability of MEF2D protein. Hep3B cells were pre‐treated with DMSO, FN, and FAK inhibitor PF562271 (10 µm) for 24 h, then incubated with 20 µg mL^−1^ cycloheximide and cell lysates collected at the indicated time points. D) Immunoblot analysis of FAK phosphorylation and MEF2D protein levels in Hep3B cells treated with FN for the indicated times, followed by incubation with PF562271 and MG132 (10 µm). E) MEF2D polyubiquitination and FAK phosphorylation in cells treated with MG132 in the presence or absence of FN and PF562271. F) Immunoblot of MEF2D protein levels in Hep3B cells expressing either MDM2 or STUB1. G) MEF2D polyubiquitination levels in *MDM2*‐ or *STUB1*‐depleted or control Huh7 cells. H) MEF2D polyubiquitination in an in vitro ubiquitination assay using purified His‐MEF2D protein. MDM2 was independently purified from 293T cells, and E1, E2 and ubiquitin (Ub) are recombinant proteins. I) Ni‐NTA pull‐down assay to measure MEF2D polyubiquitination levels in HEK293T cells transfected with Flag‐MEF2D or Flag‐MEF2D^ΔC^ (lacking AA 339–521), and HA‐MDM2 together with wild‐type (WT) ubiquitin or ubiquitin mutants containing K11R, K48R, K63R, or all lysine mutated to arginine (7KR). J) Co‐IP analysis of interaction of endogenous MEF2D and MDM2 in Hep3B cells. K) GST pull‐down analysis of the interaction between GST‐MEF2D deletion mutants and full‐length HA‐MDM2 in Hep3B cells. Schematic diagrams of MEF2D and its deletion constructs are shown. MADS, minichromosome maintenance 1 homolog, agamous, deficient, and serum response factor: TAD, transcriptional activation domain. * Marks the band corresponding to the indicated protein. L) His pull‐down analysis of His‐MDM2 deletion mutants and full‐length Flag‐MEF2D in Hep3B cells. Schematic diagrams of MDM2 and its deletion constructs are shown. BD, p53 binding domain; NES, nuclear export signal; AD, acidic domain; ZF, zinc finger. * Marks the band corresponding to the indicated protein. All immunoprecipitation and immunoblots are representative experiments of three independent replicates.

Two lines of evidence suggest that MEF2D itself may undergo feedback regulation by the pro‐metastatic niche signals: (i) the MEF2D‐regulated gene signature was increased in intrahepatic metastases compared with primary HCC tissues (Figure S1C, Supporting Information); and (ii) the protein abundance of MEF2D was elevated upon interacting with niche components in a time‐ and dose‐dependent manner (Figures 2D and 4A; Figures S3D and S8A,B, Supporting Information). Since depleting either *Itgb1* or *Itgb4* abolished these effects, we performed a chemical screen of integrin downstream signaling and identified that FAK maintained MEF2D expression (**Figure** **4**A,B; Figure S8C–E, Supporting Information). Thus, the pro‐metastatic niche regulates integrin‐FAK signaling to upregulate MEF2D expression in a positive feedback fashion.

MEF2D protein stability, but not its mRNA levels, were significantly increased by activated integrin‐FAK signaling, suggesting post‐transcriptional regulation (Figure 4C; Figure S8F, Supporting Information). Inhibiting the 26S proteasome by MG132, but not other protein degradation pathways, increased the MEF2D protein levels, suggesting that MEF2D may be targeted for degradation by the ubiquitin‐proteasome pathway (Figure S8G, Supporting Information). Consistently, integrin‐FAK signaling suppressed poly‐ubiquitination and proteasomal degradation of MEF2D (Figure 4D,E). Among five potential E3 ubiquitin ligases^[^ ^22^ ^]^ of MEF2D, we found that MDM2, but not others, significantly decreased endogenous MEF2D protein levels (Figure 4F; Figure S8H,I, Supporting Information). Conversely, depleting *MDM2*, but not *SKP2*, a reported E3 ligase of MEF2s in fibroblasts,^[^ ^23^ ^]^ increased MEF2D expression in HCC cells (Figure S8J, Supporting Information). Consistently, MDM2 interacted with MEF2D to enhance its K48‐linked poly‐ubiquitination of MEF2D in vitro and in vivo, a process mediated largely by the C terminus (amino acids (AA) 339–521) of MEF2D and the RING domain (AA 411–497) of MDM2 (Figure 4G–L; Figure S8K,L, Supporting Information). Deleting a potential MDM2 binding motif in the C terminus of MEF2D did not disrupt their interaction (Figure S8K, Supporting Information), suggesting that other motifs in the C terminus of MEF2D may be mediating its interaction with MDM2.

Functionally, MDM2 attenuated ECM‐mediated cell adhesion and NETs‐mediated cell chemotaxis, while supporting cell growth in Matrigel (Figure S8M–O, Supporting Information), a phenotype partially consistent with loss of MEF2D in these circumstances. Interestingly, integrin‐FAK signaling neither regulated expression, subcellular location, or activity of MDM2, nor affected its interaction with MEF2D (Figure S8P–R, Supporting Information). Thus, it appears that MDM2‐mediated regulation of MEF2D is not actively controlled by integrin‐FAK signaling, indicating that alternative factors are responding to the pro‐metastatic niche signals to inhibit ubiquitination and degradation of MEF2D.

### 2.5 The Deubiquitinase USP14 is Activated by Integrin‐FAK Signaling through Phosphorylation at Ser432, representing a Molecular Switch to Integrate Niche Signals for MEF2D Stabilization

**Figure 5:**
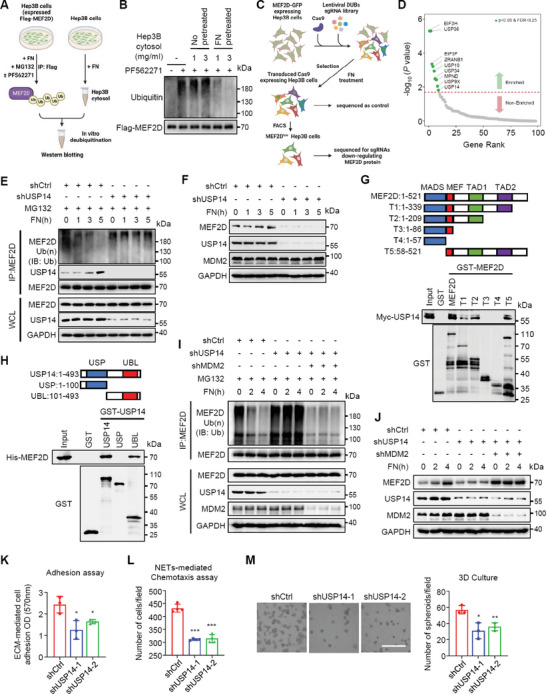
USP14 is activated by various pro‐metastatic niche signals to interact, deubiquitinate, and stabilize MEF2D, thereby promoting adhesion and chemotaxis of DCCs. A) Schematic representation of an in vitro deubiquitination assay. B) Ubiquitinated Flag‐MEF2D immunoprecipitated from Hep3B cells treated with FN (bottom coated, 10 µg mL^−1^), MG132 (10 µm), and FAK inhibitor PF562271 (10 µm). The purified Flag‐MEF2D protein was incubated with cytosolic fractions from Hep3B cells treated as indicated and analyzed by immunoblotting. C) CRISPR screening pipeline to identify DUBs for MEF2D in Hep3B cells expressing GFP‐tagged MEF2D, using a library of single‐guide RNAs (sgRNAs) targeting all DUBs in the human genome. D) Sequencing results from screen of sgRNAs targeting DUBs were sorted by the enrichment score based on the Log2 (fold change) ratio between MEF2D^low^ and control cells. The significantly enriched genes (green plots) are highlighted. E) Co‐IP analysis of MEF2D polyubiquitination, and USP14‐MEF2D interaction, in *USP14*‐depleted and control Hep3B cells that were treated with FN for the indicated times. F) Immunoblot analysis of MEF2D protein levels in *USP14*‐depleted and control Hep3B cells cultured on FN‐coated plates for the indicated times. G,H) GST pull‐down analysis of GST‐MEF2D (G) or GST‐USP14 (H) deletion mutants that were incubated with lysates of FN‐treated Hep3B cells expressing Myc‐USP14 or His‐MEF2D, respectively. Schematic diagrams of wild‐type proteins and their deletion mutants are shown. I) MEF2D polyubiquitination levels in FN‐treated Hep3B cells that were *USP14*‐depleted, or *USP14*‐ and *MDM2* double depleted. J) MEF2D protein levels were detected by immunoblotting. K) Crystal violet staining‐based quantification of *USP14*‐depleted and control Hep3B cells adhered to ECM. L) Chemotaxis assay for *USP14*‐depleted and control Hep3B cells add to the upper chambers and NETs added to the culture media in the lower chambers of transwell assay. Tumor cells:NETs = 1:5. M) 3D growth of *USP14*‐depleted and control Hep3B cells in ECM. Scale bars, 10 µm. All immunoprecipitation and immunoblots are representative experiments of three independent replicates. For all panels, * *p* < 0.05, ** *p* < 0.01, *** *p* < 0.001.

Poly‐ubiquitination of MEF2D was significantly decreased by integrin‐FAK signaling (Figure 4E), suggesting that deubiquitinase (DUB) activity may be induced by the pro‐metastatic niche signals. In line with this in vivo data, ubiquitination levels of purified MEF2D protein from cells were substantially decreased by FN‐treated, but not control cell lysate (**Figure** **5**A,B), indicating that FN may stimulate higher DUB activity toward MEF2D.

To identify potential DUBs for MEF2D, a pooled CRISPR‐screening was performed^[^ ^24^ ^]^ (Figure 5C). After FN stimulation of DUB activity, the lowest MEF2D‐expressing (MEF2D^low^) cells were sorted for sequencing and multiple DUBs were identified as potentially necessary for FN‐induced MEF2D stabilization (Figure 5D and Table S2, Supporting Information). Among these top hits, only ubiquitin‐specific protease 14 (USP14) significantly upregulated MEF2D (Figure S9A, Supporting Information). Consistently, depleting *USP14* significantly attenuated DUB activity on MEF2D induced by the niche components (Figure 5E,F; Figure S9B, Supporting Information). Moreover, mutating the enzymatic active sites of USP14^[^ ^25^, ^26^ ^]^ or its pharmacological inhibition, suppressed DUB activity toward MEF2D (Figure S9C–F, Supporting Information). Thus, USP14 is a dominant DUB for MEF2D whose activity is induced by the pro‐metastatic niche signals.

Notably, USP14 was actively recruited to MEF2D upon FN or NETs‐DNA treatment, while MDM2 constitutively interacted with MEF2D (Figure 5E; Figures S8R, S9G, Supporting Information). We further identified that the UBL domain of USP14 and TAD1 domain of MEF2D mediated their interaction (Figure 5G,H). Moreover, E3 ligase activity of MDM2 toward MEF2D was counteracted by USP14 (Figure 5I,J; Figure S9H, Supporting Information). Thus, MDM2 constitutively acts on MEF2D, whereas the pro‐metastatic niche signals actively induce USP14 to interact with, and stabilize, MEF2D. Consistently, depleting *USP14* or inhibiting its DUB activity significantly suppressed cellular adhesion, chemotaxis, and 3D growth induced by pro‐metastatic niche components (Figure 5K–M; Figure S9I–K, Supporting Information).

**Figure 6:**
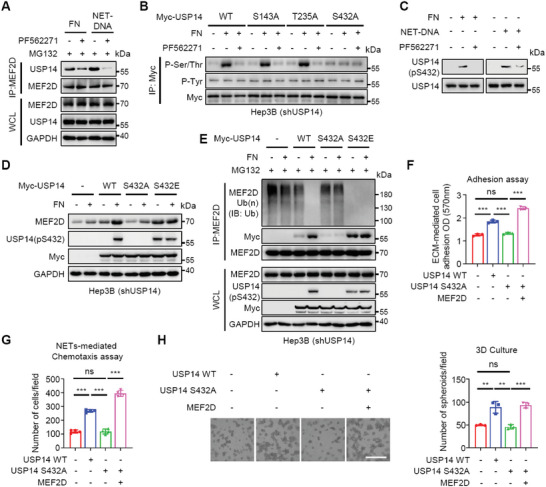
Integrin‐FAK signalling‐mediated USP14‐S432 phosphorylation triggers USP14 interaction with MEF2D, acting as a molecular switch to upregulate MEF2D and generate pro‐metastatic feedback signaling loop. A) Co‐IP analysis of MEF2D with USP14 in FN or NETs‐DNA‐treated Hep3B cells that were further incubated in the presence or absence of the FAK inhibitor PF562271 (10 µM). B) IP analysis of pSer/Thr residues in wild‐type (WT) or mutant USP14 proteins (S143A, T235A and S432A) purified from Hep3B cells that were treated with FN alone or together with PF562271. C) IP analysis of pS432 levels in endogenous USP14 proteins purified from Hep3B cells treated with FN or NETs‐DNA, together with PF562271. D) Immunoblot analysis of MEF2D protein and its pS432 levels in *USP14*‐depleted Hep3B cells expressing WT‐USP14 or USP14 mutants (S432A and S432E) proteins that were treated with or without FN. E) Co‐IP analysis of MEF2D polyubiquitination levels and UPS14 interaction with MEF2D in *USP14*‐depleted Hep3B cells expressing WT‐USP14 or USP14 mutants that were treated with or without FN. F,G) Analysis of ECM‐mediated adhesion (F) or NETs‐induced chemotaxis (G) of *USP14*‐depleted Hep3B cells expressing WT‐USP14 or USP14 S432A mutants that were rescued with ectopic expression of *Mef2d*. Tumor cells:NETs = 1:5. H) 3D growth of *USP14*‐depleted Hep3B cells reconstituted with indicated *USP14* in ECM. Scale bars, 10 µm. All immunoprecipitation and immunoblots are representative experiments of three independent replicates. For all panels, ** *p* < 0.01, *** *p* < 0.001, and ns, no significance.

To further elucidate the molecular basis for USP14 recruitment to MEF2D, we identified that integrin‐FAK signaling was essential for USP14 recruitment to MEF2D (**Figure** **6**A), which correlated with phosphorylation of USP14, but not MEF2D, on serine/threonine residues (Figure 6B; Figure S9L, Supporting Information). Among several potential phosphorylated residues on USP14 (https://www.phosphosite.org/), we found Ser432 to be the primary phosphorylated residue on the C‐terminus (Figure 6B) that sterically clashes with ubiquitin,^[^ ^25^ ^]^ as confirmed using an antibody specific to phosphorylated Ser432 (USP14 ‐pS432) (Figure 6C). Thus, USP14‐pS432 is enhanced by FAK signaling, suggesting it may be essential for its recruitment to MEF2D.

To support this notion, the phospho‐deficient USP14‐S432A, unlike wild‐type USP14 (WT) and phospho‐mimetic mutant USP14‐S432E, was not recruited to MEF2D to deubiquitinate and stabilize MEF2D (Figure 6D,E). As a result, USP14‐S432A‐expressing cells were less efficient in sustaining cellular adhesion, chemotaxis, and 3D growth induced by niche components, a phenotype that was rescued by ectopic expression of MEF2D (Figure 6F–H; Figure S9M, Supporting Information). Together, upon recognizing niche components, USP14‐S432 phosphorylation is induced via integrin‐FAK signaling to promote its association with, and deubiquitination of, MEF2D leading to its stabilization and its subsequent up‐regulation of ITGB1 and ITGB4, to amplify the pro‐metastatic feedback loop in DCCs.

### 2.6 The pUSP14‐MEF2D‐ITGB1/4 Circuit is a Prognostic Indicator in Human HCC and an Actionable Target to Inhibit Pro‐Metastatic Niche Signals and Intrahepatic Metastasis

**Figure 7:**
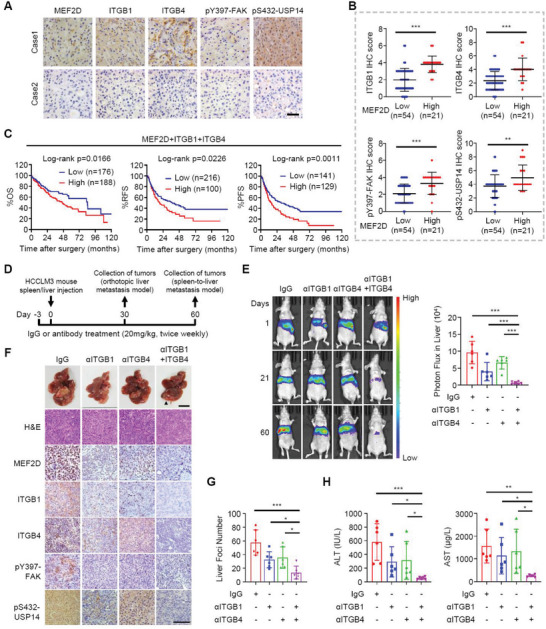
Combined targeting both ITGB1 and ITGB4 inhibits pUSP14‐MEF2D‐ITGB1/4 signaling and intrahepatic metastasis of disseminated HCC cells. A,B) IHC staining (A) of MEF2D, ITGB1, ITGB4, pY397‐FAK or pS432‐USP14 and quantitative analysis of their staining scores (B) in HCC tissues from 75 patients (Cohort II). Representative IHC images from two samples are shown. Scale bar: 50 µm. C) HCC patients were stratified for Kaplan‐Meier analysis of overall survival (OS), relapse‐free survival (RFS) and progression‐free survival (PFS) according to the co‐expression levels of MEF2D, ITGB1 and ITGB4 in tumour tissues using data from the TCGA dataset. D) Schematic of anti‐ITGB1, anti‐ITGB4, or combination treatment strategy of mice implanted with HCCLM3 cells through splenic or intrahepatic injection (n = 6). E) BLI at 1, 21, and 60 days post splenic injection of HCCLM3 cells. F) Livers resected from the spleen‐to‐liver metastasis mouse model. Tissues were photographed, fixed, and stained with haematoxylin and eosin (H&E), or immunostained for the levels of MEF2D, ITGB1, ITGB4, pY397‐FAK, and pS432‐USP14 as indicated (n = 6). Black arrow, liver metastases. Scale bar, 1 cm (top), 100 µm (bottom). G‐H) Liver metastases were counted (G) and plasma ALT and AST levels were measured (H) for mice. For all panels, * *p* < 0.05, ** *p* < 0.01, *** *p* < 0.001, and ns, no significance.

We next set out to assess the clinical relevance of the pUSP14‐MEF2D‐integrin‐FAK positive feedback loop for human HCC. Higher levels of MEF2D expression were associated with elevated immunostaining scores of ITGB1 and ITGB4, as well as activated FAK and pS432‐USP14 levels (**Figure** **7**A,B; Figure S10A, Supporting Information). Consistently, increased expression of both ITGB1 and ITGB4 were associated with shorter overall survival (OS), relapse‐free survival (RFS), and progression‐free survival (PFS) of HCC patients (Figure S10B, Supporting Information). Moreover, combined detection of MEF2D, ITGB1 and ITGB4 further identified a subpopulation of HCC patients with poorer prognosis (Figure 7C; Figure S10C, Supporting Information), suggesting that co‐detection of these three biomarkers represents a superior prognostic approach for HCC patients.

These findings are extendable to other tumor types, since MEF2D‐ITGB1‐ITGB4 signature was also co‐expressed (Figure S3G, Supporting Information) and correlated to shortened OS and disease‐free survival (DFS) in multiple human cancers (Figure S10D, Supporting Information). Thus, MEF2D‐mediated activation of the integrin‐FAK signaling is broadly observed across multiple tumor types, which may serve as valuable prognostic biomarkers across various human cancers.

In light of these findings, this signaling axis could be exploited to inhibit cancer metastasis. In this regard, a clinical trial for targeting β1‐integrin as an anti‐cancer strategy was unsuccessful.^[^ ^27^, ^28^ ^]^ However, since ITGB1 and ITGB4 executed distinct functions at different metastatic stages, a synergistic response might be observed following combined targeting of the two integrins. Indeed, the combo therapy with anti‐ITGB1 and anti‐ITGB4 antibodies markedly decreased the number of liver metastases, as compared to control or single agent treatment (Figure 7D–G; Figure S10E, Supporting Information). The liver metastatic nodules from the combo group showed lower levels of FAK‐Y397 and USP14‐S432 phosphorylation, and reduced expression of the MEF2D‐ITGB1/4 axis (Figure 7F). Due to reduced tumor burden, plasma alanine aminotransferase (ALT) and aspartate aminotransferase (AST) levels were markedly lower in the combo group (Figure 7G,H). In addition, these treatment strategies did not affect body weight (Figure S10F, Supporting Information). Together, blocking both ITGB1 and ITGB4 may be an improved strategy to eradicate DCCs, thereby preventing intrahepatic metastasis and post‐surgery relapse for patients with HCC.

## 3 Discussion

**Figure 8:**
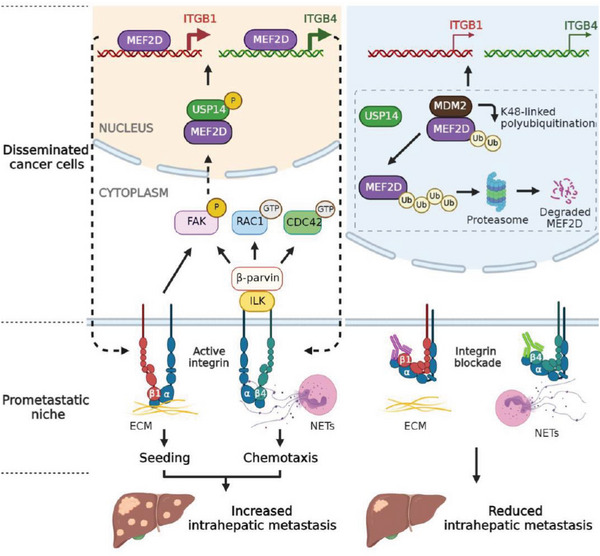
Schematic summary of the main findings in this study.

Although the invasion‐metastasis cascade has been extensively studied, relatively less is known on how DCCs adapt to the distal tissue microenvironment for dissemination.^[^ ^1^ ^]^ Our data support a model that after extravasation, DCCs rely on a core transcription factor MEF2D to upregulate ITGB1 as a molecular hook in the pro‐metastatic niche. Later in the colonization stage, DCCs act again through MEF2D to upregulate ITGB4, thereby coordinating sequential niche signals from the ECM and NETs to activate the FAK pathway and synergistically support intrahepatic metastasis (**Figure** **8**). Blocking this DCC‐niche crosstalk mechanism may represent a promising target to inhibit intrahepatic spreading of DCCs and prevent post‐surgery recurrence for HCC patients.

As an essential regulator of DCC fitness in the pro‐metastatic niche, MEF2D functions independent of regulating the EMT, a process observed in colorectal cancer cells detaching from the primary lesions^16^. This difference could be due to metastatic stage‐specific or cancer type‐specific functions of MEF2D. Here, MEF2D‐driven DCC colonization was linked to NETs, the highly enriched niche components in the liver, an organ that is highly susceptible to cancer metastasis.^[^ ^10^, ^11^, ^29^ ^]^ Additionally, except for ITGB1/4, MEF2D also regulates α integrin subunits that may interact with β integrins to form heterodimers,^[^ ^6^ ^]^ although it remains unclear which may be the dominant heterodimers in DCCs.

Notably, ITGB4, a key target of MEF2D, functions as a previously unrecognized receptor for NETs‐DNA in the pro‐metastatic niche. NETs are webs of chromatin‐DNA complexes released by necrotic neutrophils to promote microbial defense and also promotes cancer metastasis to the liver and lung.^[^ ^8^, ^30^ ^]^ We found that NETs are enriched in the metastasized tumor and the adjacent liver tissues, but not in the liver at the early DCCs seeding stage.^[^ ^31^ ^]^ This is consistent with a role of ITGB4 in engaging NETs‐DNA to maintain metastatic colonization, but not the seeding of DCCs. Moreover, this process is independent of another NETs‐DNA sensor, CCDC25.^[^ ^8^ ^]^ Thus multiple receptors, such as CCDC25 and ITGB4, may be utilized to mediate diverse functions of NETs in different biological processes.^[^ ^32^ ^]^

Our present study also elucidates a niche‐responsive ubiquitination‐dependent switch that dictates MEF2D protein stability. By functional CRISPR‐screening, we identified USP14, a mediator of liver metastasis of pancreatic cancer,^[^ ^33^ ^]^ as a key regulator of MEF2D deubiquitination and stabilization. Importantly, USP14 is phosphorylated at Ser432 by the integrin‐FAK pathway to promote is recruitment to MEF2D. Therefore, a pUSP14‐MEF2D‐ITGB1/4 signaling axis represents a novel positive feedback loop to promote pro‐metastatic signaling. However, FAK and several downstream kinases could not directly phosphorylate USP14 in vitro (data not shown),^[^ ^20^, ^26^ ^]^ indicating there is a kinase in this loop yet to be identified.

Finally, our findings underscore that blocking the ability of DCCs to sense these key niche signals may be a promising therapeutic modality to inhibit caner metastasis. This notion is of paramount importance for HCC, where intrahepatic metastasis occurs in 40%−60% patients, leading to disease relapse with few effective treatments.^[^ ^11^, ^34^ ^]^ Although multiple components in the USP14‐MEF2D‐integrins feedback loop could be theoretically targeted, their small‐molecule inhibitors exhibit toxicity and adverse side effects in humans.^[^ ^35^ ^]^ On the other hand, antibodies blocking integrins are clinically applicable to treat inflammatory bowel diseases and cancer.^[^ ^27^, ^28^, ^36^ ^]^ To this end, we observed that combination treatment targeting both ITGB1 and ITGB4 provided superior anti‐metastatic efficacy. Since overexpression of the MEF2D‐ITGB1/4 axis correlates with inferior outcomes in multiple cancer types including HCC, this therapeutic strategy might be broadly utilized to inhibit cancer metastasis.

## 4 Experimental Section

### Enrichment Analysis of Potential Transcription Factors

Motif enrichment analysis was performed to identify potential TFs that might regulate signaling pathways in the liver pro‐metastatic niche. To this end, 102 gene signatures of key pro‐metastatic niche‐related signaling pathways were collected from the literature^[^ ^37^, ^38^, ^39^ ^]^ and from MSigDB,^[^ ^40^ ^]^ including chemokines, focal adhesion, integrin signaling, FAK pathway, growth factors, Hippo pathway, and the HIF pathways. TF motif enrichment was carried out among the promoter regions of 2290 genes in collected signatures by script “findMotifs.pl” of homer.^[^ ^41^ ^]^ Differential gene expression analysis was performed with TPM values of TCGA‐LIHC RNA‐seq datasets obtained from the UCSC Xena platform^[^ ^42^ ^]^ by the ggpubr package.^[^ ^43^ ^]^ GSVA^[^ ^44^ ^]^ and corrplot package^[^ ^45^ ^]^ were used to calculate the correlation of enriched transcription factors and liver pro‐metastatic niche signatures.

### Human Samples

Human HCC specimens were obtained from 3 cohorts. Cohort I (Figure S2D, Supporting Information) included 145 patients from Southwest Hospital, as previously described.^[^ ^12^ ^]^ Cohort II (Figure 7A) were purchased from Outdo Biotech (HlivH150CS05), comprising 75 HCC patients (58 men and 17 women, 25–73 years old) undergoing surgery. Cohort III (Figure S10A, Supporting Information) were derived from Daping Hospital, comprising 100 HCC patients (87 men and 13 women, 29–79 years old) undergoing surgery. Clinical diagnosis was based on the World Health Organization Tumor Classification. This study was approved by the Ethics Committee of Daping Hospital, the Third Affiliated Hospital of Army Medical University (No.2018‐58), and all specimens were collected with informed consent.

### Cell Culture

Human HCC cell lines (Hep3B, Huh7, HepG2 and PLC/PRF/5), NIH/3T3 mouse fibroblasts, and J774A.1 mouse macrophages were purchased from the American Type Culture Collection (Manassas, VA). Other human HCC cell lines (HCCLM3, MHCC97H) were purchased from Procell Life Science & Technology (Wuhan, China). All cell lines were validated by short tandem repeat analysis. Mouse T cells, neutrophils, NK cells, and endothelial cells were isolated using the Pan T Cell Isolation Kit (Miltenyi Biotec, 130‐095‐130), Ly‐6G^+^ microbeads (Miltenyi Biotec, 130‐120‐337), CD49b^+^ MicroBeads (Miltenyi Biotec, 130‐115‐818), and CD31^+^ MicroBeads (Miltenyi Biotec, 130‐097‐418), respectively. All cell lines were routinely tested and demonstrated to be negative for mycoplasma contamination. Cells were expanded and frozen to allow revival every 3–4 months, and were routinely cultured in DMEM or RPMI‐1640 media containing 10% heat‐inactivated fetal calf serum (Biological Industries, Kibbutz Beit Haemek, Israel) at 37 °C/5% CO_2_. To generate MEF2D, ITGB1, and ITGB4 knockout HCC cell lines, CRISPR/Cas9 gene editing was applied using commercial plasmids carrying encoded green fluorescent protein (EGFP) proteins (Santa Cruz Biotechnology, Dallas, TX). Each CRISPR/Cas9 knockout product consisted of a mixture of 3 plasmids, each encoding the Cas9 nuclease and a target gene–specific 20‐nucleotide guide RNA (gRNA) designed for maximum KO efficiency. gRNA sequences were derived from the GeCKO (v2) library and direct the Cas9 protein to induce a site‐specific double‐strand break in the genomic DNA. CRISPR gRNA sequences are listed in Table S4 (Supporting Information). HCC cells were transfected using Effectene Transfection Reagent (Qiagen, Venlo, The Netherlands). The transduced cells were selected by FACS using a BD FACS Aria II instrument according to GFP positivity to obtain knockout cells.

### Mouse Models and Animal Treatments

BALB/c nude mice (male, 6 weeks old) were obtained from Beijing HFK Bioscience, China. *Mef2d^fl/fl^* mice and *Alb‐cre* mice on a C57BL/6 background were purchased from Cyagen Biosciences. The mice were maintained in pathogen‐free conditions. All procedures were approved by the Army Medical University Medical Ethics Committee (Reference number: SYXK 20170002) and conformed to the National Institutes of Health guidelines on the ethical use of animals. Otherwise indicated, no exclusions or no criteria were set for animal assays. DNase I treatment was performed as previously described.^[^ ^46^ ^]^ Briefly, mice were injected i.p. with DNase I (5 mg k^−1^ g) 2 h before HCC cell injection, followed by daily injection of the DNase I for 9 days, and subsequently maintenance injections twice a week.

### Spleen‐to‐Liver Metastatic Seeding and Colonization Model

A spleen‐to‐liver metastasis model was established as previously described,^[^ ^16^ ^]^ HCCLM3 cells were injected into the spleen of nude mice at the dose of 2 × 10^6^ cells per mouse. For bioluminescence imaging (BLI), mice were injected i.p. with D‐luciferin (150 mg k^−1^ g) and then imaged using an IVIS Spectrum instrument (PerkinElmer) to monitor the systemic dissemination of tumor cells. Metastatic burdens were also measured at indicated time points using BLI. BLI images were analyzed using Living Image Software v.4.20. Liver metastatic tumor burden was measured by the photon flux of the BLI signal within a region that corresponds to the liver which was manually outlined. Alternatively, HCCLM3 cells were labeled with CMFDA C2925 (Thermo Fisher Scientific, Carlsbad, CA) and injected as described above. After 48 h, the mice were sacrificed, and livers were removed and fixed with 10% paraformaldehyde at 4 °C. The livers were dissected coronally and were embedded in 4% agarose. Vibratome sections (100 mm thick) were prepared and mounted on glass slides. Immunofluorescence staining was carried out on liver sections with mouse CD31 antibody to mark microvascular endothelial cells. CMFDA positive cancer cells in the sections were detected using a fluorescent microscope and counted. To prevent the intrahepatic metastasis, mice were preconditioned twice a week by i.p. injection of ITGB1 and ITGB4‐neutralizing antibody (20 mg k^−1^ g) 3 days before HCC cell implantation. Six mice per treatment group were included. No randomization or blinding was used in the tumor metastasis experiments. At the end of the experiments, the mice were weighed and euthanized. Plasma was collected into EDTA‐containing tubes, in which ALT and AST were tested using Mouse Alanine Aminotransferase (ALT) ELISA Kit (Abcam, ab285263) and AST SimpleStep ELISA Kit (Abcam, ab263882), respectively. Liver metastases were quantified and minced for further analysis.

### Orthotopic Injection and Intrahepatic Metastatic Model

To better mimic the intrahepatic metastasis of the orthotopic HCC,^[^ ^14^ ^]^ Hepa1‐6 (1 × 10^6^) or H22 (1 × 10^6^) murine HCC cells, or HCCLM3 human HCC cells (2 × 10^6^), were mixed with 25 µL Matrigel Basement Membrane Matrix (ECM) (BD Biosciences, Franklin Lakes, NJ) and injected subcapsular into the paramedian area of the lower surface of the left hepatic lobe of C57BL/6 mice or BALB/c mice, or nude mice, respectively. The puncture site was closed with acrylic glue (COMPONT, Beijing, China). After one month the mice were sacrificed, and tumors were carefully dissected.

### Diethylnitrosamine (DEN)‐Induced HCC Mouse Model

A single dose of 25 mg k^−1^ g of DEN was injected i.p. into 15‐day old C57BL/6 *Alb‐cre^+^*;*Mef2d^fl/fl^* or *Alb‐cre^−^*;*Mef2d^fl/fl^* male mice. Mice were sacrificed at 11 or 14 months. Tumor tissue was collected and processed for immunofluorescence analysis.

### Bioinformatics Processing of the ChIP‐Seq and RNA‐Seq Datasets

Poly (A)(+) messenger RNA (mRNA) was enriched by Oligo dT and then quantified and quality controlled by an Agilent Bioanalyzer 2100 system. After cDNA library construction, the library preparations were sequenced on BGISEQ500 platform (BGI). The RNA‐seq data are available at the GEO database (accession number: GSE224425). Publicly available ChIP‐seq and RNA‐seq datasets were downloaded from GEO database with accession numbers: GSE37123, GSE74656, GSM2425733, GSM2617031, GSM4011081, GSM4851386, GSM2425731, GSM2617029, GSM3357691, GSM5215167, GSM5215168. The low‐quality reads of both ChIP‐seq and RNA‐seq were filtered by fastp^[^ ^47^ ^]^ (version 0.20.1). The cleaned reads of ChIP‐seq were mapped to the human reference genome (hg38) using Bowtie2^[^ ^48^ ^]^ (version 2.1.0) with default parameters. MACS2 (version 2.1.1)^[^ ^49^ ^]^ was implemented to call peaks. The mapped reads were normalized and visualized using deepTools^[^ ^50^ ^]^ (version 3.4.1) and Integrative Genomics Viewer, respectively. The cleaned reads of RNA‐seq were aligned against the hg38 transcriptome using HISAT2^[^ ^51^ ^]^ (version 2.1.0) and quantitated with featureCounts.^[^ ^52^ ^]^ Gene expression was normalized to TPM with in‐house developed scripts. Low expression genes were filtered, and raw count matrix was used for differential expression analysis using edgeR^[^ ^53^ ^]^ (version 3.28.1). Differential expressed genes (DEGs) were set as FDR < 0.05 and |log2 FoldChange| > 0.58. The clusterProfiler^[^ ^54^ ^]^ package in R (version 3.6.1) was implemented to perform GO and KEGG pathway enrichment analyses of DEGs.

### Immunohistochemistry and Immunofluorescence (IF)

Immunohistochemistry (IHC) staining was conducted as previously described.^[^ ^14^ ^]^ Briefly, formalin‐fixed paraffin‐embedded specimens were cut into 2–5 µm sections, which were deparaffinized, rehydrated, and blocked with 3% H_2_O_2_ for 15 min. Tissue antigens were retrieved by heating the sections in a pressure cooker for 150 s. After cooling to room temperature, each section was incubated with primary antibody (Table S3, Supporting Information) at 4 °C overnight, and then with goat anti‐rabbit secondary antibody (Table S3, Supporting Information) and mouse immunoglobulins (DAKO, Copenhagen, Denmark). For visualization, 1%–5% 3,3′‐diaminobenzidine tetrahydrochloride was used as a chromogen. After washing with water, the sections were counterstained with hematoxylin. In all assays, negative control slides with the primary antibody replaced with phosphate‐buffered saline (PBS) were included. All immunostained sections were independently evaluated by two pathologists blinded to the patients’ clinical status, with interobserver discrepancy being less than 10%. In the case of discrepancy, a consensus interpretation was reached with a third pathologist. The quantification method was based on a multiplicative index of the average staining intensity (score 0–3) and extent of staining (score 0, 0%; 1, 0%–25%; 2, 25%–50%; 3, 50%–75%; 4, 75%–100%) in the cores, yielding a staining index ranging from 0 (no staining) to 12 (extremely extensive and/or strong staining). For IF analysis, tissue sections were permeabilized with PBS‐T (containing 0.25% Triton X‐100) for 30 min and blocked with 5% goat serum in PBS for 60 min. The primary antibody diluted 1:25‐200 in TBS‐T (containing 0.1% Tween‐20) were incubated at 4 °C overnight. IF staining was performed with appropriate Alexa Fluor 488, Alexa Fluor 594, or Alexa Fluor 647 secondary antibodies (Invitrogen, diluted 1:500). Coverslips were mounted on slides using anti‐fade mounting medium with DAPI. Images were acquired using a ZEISS 880 Microscope.

### Plasmids, Short Hairpin (sh) RNAs, and Lentiviruses

Eukaryotic expression vectors encoding Flag‐, Myc‐, His‐, GST‐tagged or untagged proteins were generated by inserting PCR‐amplified fragments into the pcDNA3.1 vector. Prokaryotic plasmids encoding GST‐ or His‐fusion protein were constructed in pGEX‐KG and pET28a, respectively. Promoter and enhancer luciferase reporters for *Itgb1* and *Itgb4* genes were generated by inserting promoter fragments PCR‐amplified from genomic DNA into the pGL3‐Basic vector. Mutant Flag‐, Myc‐, GST‐ and His‐tagged proteins as well as the luciferase reporters were generated by PCR. To generate MEF2D‐, or ITGB1‐overexpressing stable cell lines, full‐length human cDNAs were cloned into pLVX. To generate CRISPR knockout cell line, gRNA sequences were cloned into lenti dCAS‐VP64 Blast. Lentiviral shRNA vectors were constructed by cloning shRNA fragments into pLKO.1. The cDNA target sequences of shRNAs for MEF2D, ILK, MDM2, STUB1, SKP2, and USP14 are listed in Table S4 (Supporting Information). Lentivirus was produced by cotransfection of HEK293T cells with recombinant lentiviral vectors and lentiviral packaging plasmids pMD2.G, pRSV‐Rev, and pMDLGpRRE using a calcium phosphate precipitation‐based method. After 48 h of transfection, supernatant containing virus particles was harvested and concentrated.

### Quantitative Reverse Transcription Polymerase Chain Reaction (qRT‐PCR)

Total RNA was extracted from cells using RNAiso Plus (Takara, Mountain View, CA) and reverse‐transcribed with the PrimeScript RT Reagent kit (Takara, Mountain View, CA) according to the manufacturer's instructions. Quantitative PCRs were run with SYBR premix Ex Taq (Takara, Mountain View, CA) on a CFX96 Real Time PCR Detection System (Bio‐Rad, Hercules, CA). Relative expression was calculated by the comparative Ct method. The primers used for qPCR analysis are listed in Table S4 (Supporting Information).

### Chromatin Immunoprecipitation

Chromatin immunoprecipitation (ChIP) assays were conducted using the EZ ChIP Kit (Millipore, Bedford, MA) according to the manufacturer's instructions. In brief, cells were cross‐linked with 37% formaldehyde, pelleted, and resuspended in lysis buffer. The cells were sonicated and centrifuged to remove insoluble material. Supernatants were collected and incubated overnight with indicated antibodies (Table S3, Supporting Information) and Protein G Agarose. The antibody‐Protein G‐agarose conjugates were washed, and the precipitated chromatin complexes were collected, purified, and de‐crosslinked with 200 mM NaCl at 65 °C overnight. The DNA was purified using spin columns and the collected DNA fragments were quantified by qRT‐PCR. The samples were analyzed by qPCR with primers listed in Table S4 (Supporting Information).

### Luciferase Reporter Assay

Luciferase reporter assays were performed as previously described.^[^ ^14^ ^]^ In brief, cells were seeded in 24‐well plates and were transfected with 1 µg of truncated or mutated *Itgb1* or *Itgb4* enhancer and/or promoter luciferase reporter vectors, 0.5 µg of empty vector or *Mef2d* expression vector, and 0.1 µg of β‐galactosidase reporter. The cells were harvested 48 h post transfection using lysis buffer. Luciferase activity was measured using the Dual‐Luciferase Reporter Assay System (Promega, Madison, WI) according to the manufacturer's instructions on a Varioskan LUX instrument (Thermo Fisher Scientific, Carlsbad, CA).

### Adherence Assay

Adherence of disseminated cells to the ECM, fibronectin (FN) or FN+NETs were evaluated using 96‐well plates that were pre‐coated with 8% ECM, 10 µg mL^−1^ fibronectin (Roche), or FN containing 10 µg mL^−1^ NETs overnight at 37 °C. Various cells (1 × 10^5^ cells in 100 µL) pretreated with or without inhibitor were suspended in DMEM serum‐free medium and allowed to adhere to the plate bottom for 1–3 h at 37 °C. After removing non‐adherent cells by gently washing with PBS three times, the adhered cells were fixed in 4% paraformaldehyde for 20 min at room temperature and stained with crystal violet overnight at 4 °C. Cell adherence was counted as cells per field of view under phage‐contrast microscopy or OD per well.

### Transwell Migration, Invasion, and Chemotaxis Assays

The migration, invasion, and chemotaxis of DCCs were evaluated by transwell assays. Hanging cell culture inserts (8 µm) were purchased from Millipore and placed in 24‐well plates. Cells were seeded in the upper chambers (5 × 10^5^ for migration and invasion assays and 1×10^5^ for chemotaxis assay). For migration assays, 100 µL serum‐free medium was added to the upper chambers, and 800 µL medium containing 20% FBS was added to the lower chambers as a chemo‐attractant. For invasion assay, the upper chambers were pre‐coated with 50 µL of 1 mg mL^−1^ ECM before cell seeding. For chemotaxis assay, the upper and lower chambers were filled with normal culture medium (10% FBS), and lower chambers with indicated cell types (5 × 10^5^). Time of hanging culture was 16–24 h for migration and chemotaxis assays and 48–72 h for invasion assays. The cells on the upper surface of the filter were removed carefully with a cotton swab. The cells that migrated/invaded to the underside of the filter were fixed in methanol, stained with 0.25% crystal violet, and counted. For each transwell filter, 3–4 random fields were calculated under microscope with 100× magnification.

### 3D culture in ECM

3D cultures of HCC cells were carried out in 96‐well plates. In brief, 40 µL Matrigel was added to the wells and spread evenly. After the Matrigel solidified at 37 °C, 1000 cells were resuspended in 200 µL of assay medium containing 4% Matrigel, with or without 5 ug mL^−1^ NET‐DNA, and added on top of the solidified Matrigel and cultured for 5 days.

### Immunoblot Analysis

Immunoblot analysis was performed using standard protocols. Briefly, cells were lysed in lysis buffer (Thermo Fisher Scientific, Carlsbad, CA) containing a protease inhibitor cocktail (Roche Diagnostics, Indianapolis, IN). The protein concentration in the cell lysates were determined with a Bicinchoninic Acid Assay kit (Thermo Fisher Scientific, Carlsbad, CA), and 20 µg of total protein was resolved by sodium dodecyl sulfate‐polyacrylamide gel electrophoresis (SDS‐PAGE) and transferred to nitrocellulose membrane. Membranes were blocked in 5% skim milk at room temperature for 1 h and then incubated with appropriate antibodies (Table S3, Supporting Information) at 4 °C overnight. The membrane was washed with PBS‐T and incubated with peroxidase‐conjugated secondary antibodies, and protein bands were visualized by chemiluminescence (Thermo Fisher Scientific, Carlsbad, CA) using a Gel Doc imaging system (Bio‐Rad, Hercules, CA). Densitometric analysis of protein bands was conducted using ImageLab 4.0 software (Bio‐Rad, Hercules, CA).

### Co‐immunoprecipitation (Co‐IP) and GST/His Pull‐Down

Cells were transfected with plasmids using Effectene Transfection Reagent, lysed in 500 µL of immunoprecipitation lysis buffer (Thermo Fisher Scientific, Carlsbad, CA), and immunoprecipitated with the indicated antibodies (Table S3, Supporting Information) and Protein A/G Magnetic Beads (Thermo Fisher Scientific, Carlsbad, CA) at 4 °C overnight. The beads were washed with lysis buffer three times and eluted in SDS sample buffer. The eluted immunocomplexes were separated by SDS‐PAGE, followed by immunoblotting with indicated antibodies (Table S3, Supporting Information) as described above. To detect endogenous protein interactions, tumor cells were lysed in 500 µL of immunoprecipitation lysis buffer and immunoprecipitated with the indicated antibodies (Table S3, Supporting Information) and Protein A/G Magnetic Beads. The beads were washed with lysis buffer three times and eluted in SDS sample buffer. The eluted immunocomplexes were separated by SDS‐PAGE, followed by immunoblotting as described above. For His or GST pull‐down assays, His‐ or GST‐fusion proteins were expressed and purified according to the manufacturer's instructions (Thermo Fisher Scientific). His‐ or GST‐fusion proteins were expressed in *Escherichia coli* (BL21). After induction with 0.5 mM isopropyl‐β‐D‐thiogalactoside at 20 °C for at least 20 h, *E. coli* cells were collected, resuspended in lysis buffer, and sonicated. The lysates were centrifuged, and supernatants were loaded on Ni‐NTA beads (Thermo Fisher Scientific, Carlsbad, CA) or glutathione magnetic beads (Thermo Fisher Scientific, Carlsbad, CA) balanced with lysis buffer at 4 °C for 1 h. The beads were collected and washed with lysis buffer three times. Cell lysates were incubated with GST‐ or His‐fusion protein bound to GST beads/Ni‐NTA beads, at 4 °C overnight. After washing, the adsorbed proteins were eluted in SDS sample buffer. The eluted proteins were separated by SDS‐PAGE, followed by immunoblotting as described above.

### In Vitro Ubiquitination Assay

Varying amounts (0.01, 0.1, 1, and 10 µg) of purified His‐MEF2D protein was incubated in 30 µL reaction mixture containing 50 mM Tris, pH 7.5, 5 mM MgCl_2_, 2 mM NaF, 2 mM ATP, 10 mM Okadaic acid, 1 mM DTT, 0.1 µg E1, 0.2 µg E2, and 1 µg HA‐ubiquitin. Approximately 200 ng of bacterially expressed and purified GST‐MDM2 was added and reactions were incubated at 37 °C for 1 h before being stopped by addition of SDS‐PAGE loading buffer.

### Production and Purification of Recombinant ITGB1 and ITGB4 Proteins

Full‐length ITGB1 and ITGB4 was cloned into pcDNA3.1 expression vector (Novagen) with a His‐tag fusion at the C terminus. The plasmids were transfected into HEK293T cells. After 48 h, cells were pelleted and lysed in pull‐down lysis buffer (Thermo Scientific, 1858601). The His‐tagged protein was isolated from the supernatants using the HisPur Ni‐NTA Purification Kit (Thermo Scientific, 88229).

### Purification of NETs and NETs‐DNA

NETs were isolated from primary mouse neutrophils in bone marrow as described previously.^[^ ^8^ ^]^ Neutrophils were treated with 500 nM PMA for 4 h. After removal of the supernatant, NETs adhered at the bottom were washed by pipetting 2 mL of cold PBS and were centrifuged at 1000 × g at 4 °C for 10 min. Cell‐free supernatant containing NETs (DNA‐protein complex) were collected. The DNA concentration of NETs were measured by spectrophotometry and the NETs were used for further experiments. Isolated NETs were fragmented with a VCX130 sonicator to the length of 200–500 bp and subsequently purified using a MicroElute DNA Clean Up Kit (OMEGA, D6296) and biotinylated using Biotin 3′ End DNA Labelling Kit (Thermo Scientific, 89818) according to the manufacturer's instructions.

### Biotinylated NETs‐DNA Pull‐Down

NETs (DNA‐protein complex; 200 µg) were incubated with DNA‐binding Dynabeads (4 mg mL^−1^, Thermo Scientific, 37002D) at room temperature for 1 h and treated with PBS or proteinase K (0.5 µg mL^−1^, Thermo Scientific) for 4 h at 56 °C. After centrifugation and extensive washing, the Dynabeads‐NETs complexes were incubated with 0.5 µg recombinant His‐ITGB1 or His‐ITGB4 protein at room temperature for 1 h, followed by washing four times with IP lysis buffer. Bound protein was eluted with 1X SDS loading buffer by boiling for 5 min and resolved by SDS‐PAGE and immunoblotted with anti‐His antibody. In indicated experiments, 200 µg of NETs (DNA‐protein complex) was incubated with the Biotin‐XX sulfosuccinimidyl ester (Thermo Scientific, F‐20650) on ice for 30 min followed by incubation with streptavidin‐microbeads at room temperature for 1 h. After centrifugation and extensive washing, the Dynabeads‐NETs complex was treated with PBS or DNase I (0.25 mg mL^−1^, Roche, 11284932001) for 1 h at 37 °C and incubated with 0.5 µg recombinant His‐ITGB1 or His‐ITGB4. The beads were washed four times with IP lysis buffer. Bound protein was eluted with 1X SDS loading buffer by boiling for 5 min and resolved by SDS‐PAGE and immunoblotted with anti‐His antibody.

### Oxidative dsDNA Pull‐Down

Three heterologous double‐stranded DNA oligonucleotides^[^ ^8^ ^]^ (Oligo 1: 5′‐CGGGTGTCGGGGCTGGCTTAACTATGCGGCATCAGAGCAGATTGTACTGAGAGTGCACCATATGCGGTGTGAAATACCGCACAGATGCGT‐3′; Oligo 2: 5′‐TACAGATCTACTAGTGATCTATGACTGA TCTGTACATGATCTACATACAGATCTACTAGTGATCTATGACTGATCTGTACATGATCTACA‐3′; Oligo 3: 5′‐GGGCTAC CGTCAAGTAAGATGCAGATACGGAACACAGCTGGCACAGTGGTAGTACTCCACTGTCTGGCTGTACAAAAACCCTCGGGATCT‐3′) were dissolved in sterile H_2_O at a concentration of 20 ng µL^−1^ and were irradiated with UV‐C light at 250 mJ cm^2^ using a SCIENTZ 03‐II hybridization oven. The relative 8‐OHdG content in the DNA was quantified with the 8‐OHdG ELISA Kit (E‐EL‐0028c, Elabscience). The oxidative DNA pull‐down was performed as follows: 2 µg recombinant His‐ITGB4 protein was incubated with 600 ng oxidative or unmodified DNA in 400 µL IP lysis buffer at room temperature for 1 h. The protein‐DNA complex was then incubated with 50 µL streptavidin‐agarose beads at room temperature for another 1 h. The beads were then washed three times with IP lysis buffer and separated by gradient gel electrophoresis followed by immunoblotting with anti‐His antibody.

### Electrophoretic Mobility Shift Assay

The electrophoretic mobility shift assay (EMSA) assay was performed according to the manufacturer instructions using the LightShift Chemiluminescent EMSA Kit (Thermo Scientific, 20148). Biotinylated 8‐OHdG‐DNA (1 ng) and 2 µg of isolated membrane protein were incubated in the EMSA binding buffer for 20 min at room temperature. The samples were applied to a 4% PAGE gel in 0.5X TBE (Tris‐borate‐EDTA) buffer for 1.5 h at 100 V. The resolved reactions on the gel were transferred to a Nylon membrane for 1 h at 380 mA and the protein‐DNA‐binding complex was crosslinked to the membrane. The membrane was incubated with blocking solution at room temperature for 30 min to block non‐specific binding followed by incubation with stabilized streptavidin‐horseradish peroxidase at room temperature for 30 min. The membrane was then washed four times with wash solution and once with substrate equilibration buffer. The presence of a band shift was assessed using chemiluminescent substrate solution.^[^ ^8^ ^]^

### In Vitro Deubiquitination Assay

Hep3B cells were treated with or without PF562271 (FAK inhibitor, 10 µM) in the presence of FN (10 µg mL^−1^) and MG132 (20 µM) for 4 h to induce MEF2D ubiquitination. Cytosolic factions were isolated from cells and immunoprecipitated with anti‐MEF2D antibody‐coupled agarose. The pelleted complexes were then incubated with the cytosolic fractions from Hep3B cells that were treated with or without FN for 30 min at 37 °C. The reaction was stopped by adding 1X SDS loading buffer, followed by SDS‐PAGE and immunoblotting as described above. For deubiquitination assay using purified USP14 protein, Hep3B cells were transfected with or without MDM2 to induce MEF2D ubiquitination. Cytosolic fractions were immunoprecipitated with anti‐MEF2D antibody‐coupled agarose. The pelleted complexes were then incubated with purified GST‐USP14 protein derived from *E. coli* (BL21) in 40 µL reaction buffer (50 mM Tris HCl, pH 7.3, 1 mM MgCl_2_, 50 mM NaCl, and 1 mM DTT) for 3 h at 37 °C, and the reaction was stopped by adding 1X SDS loading buffer, followed by SDS‐PAGE and immunoblotting as described above.

### Pooled sgRNA Library Design and Construction

Cloning the sgRNA library was performed as previously described.^[^ ^55^ ^]^ A targeted library to include 98 DUBs genes with 4 guides per gene was designed. Guides were from the Brie sgRNA library,^[^ ^56^ ^]^ and the pooled oligo library was designed to match the vector backbone. Oligos were PCR amplified and cloned into the LentiCRISPR v2 backbone by Gibson assembly as previously described.^[^ ^55^ ^]^ All oligos included in the library and primer sequences are listed in Table S4 (Supporting Information).

### Pooled CRISPR Screen Pipeline and Analysis

A lentiviral reporter vector pLenti‐hygro‐CMV‐DsRed‐IRES‐MEF2D‐EGFP was generated to encode both DsRed and MEF2D‐EGFP proteins. The reporter system was stably integrated into Hep3B cells and selected with hygromycin to obtain stable clones. The MEF2D‐GFP‐expressing Hep3B cells were transduced with the sgRNA library via lentiviral transduction and cultured at a density of 1 million cells mL^−1^ continually to maintain a library coverage of at least 1000 cells per sgRNA. Eight days after the transduction, cell subpopulations were treated by FN (10 µg mL^−1^) for 12 h and then sorted by FACS based on MEF2D protein levels relative to DsRed (EGFP/DsRed fluorescent intensity). Genomic DNA was collected from each subpopulation and the sgRNA‐encoding regions were then amplified by PCR and sequenced on an Illumina MiniSeq using custom sequencing primers. By analyzing the sequencing data, the frequencies of cells expressing different sgRNAs in control and MEF2D^low^ subpopulation was.quantified To identify hits from the screen, the MAGeCK software to quantify and test for guide enrichment was used.^[^ ^57^ ^]^ Abundance of guides was first determined using the MAGeCK “count” module for the raw fastq files. For the targeted libraries, the constant 5′ trim was automatically detected by MAGeCK. To test for robust guide and gene‐level enrichment, the MAGeCK “test” module was used with default parameters. This step included median ratio normalization to account for varying read depths. The non‐targeting control guides to estimate the size factor for normalization was used, as well as to build the mean‐variance model for null distribution, which was used to find significant guide enrichment. MAGeCK produced guide‐level enrichment scores for each direction (positive and negative) which were then used for alpha‐robust rank aggregation to obtain gene‐level scores. The *P* value for each gene was determined by a permutation test, randomizing guide assignments and adjusted for false discovery rates by the Benjamini‐Hochberg method. The Log2 (fold change (FC)) was calculated for each gene, defined throughout as the median Log2 (FC) for all guides per gene target. Where indicated, Log2 (FC) was normalized to have a mean of 0 and S.D. of 1 to obtain the Log2 (FC) z‐score. sgRNA and gene level enrichment and raw count files can be found in Table S2 (Supporting Information).

### Statistical Analysis

GraphPad Prism software (version 8) was used for all other statistical analyses. Randomization was used for experimental grouping. Means between two groups were compared by Student's t‐test (unpaired, two‐tail) (n > 10) or Mann‐Whitney test (n < 10), and multiple comparisons were made by analysis of variance (ANOVA). Survival data were presented as Kaplan‐Meier survival curves, and differences between groups were evaluated using the log‐rank test. Pearson correlation analysis was performed to determine correlations among groups. For all statistical analyses *P* < 0.05 was considered statistically significant. Otherwise indicated in the figure legends, all data were presented as the mean ± SD.

## Conflict of Interest

The authors declare no conflict of interest.

## Author Contributions

J.X., N.Z., A.D. share co‐first authorship. J.X., N.Z., A.D., J.L., M.L., Y.W., M.L., L.Y., X.L., L.W., and Q.L. performed experiments. X.L., Z.Q., T.W. and L.W. analyzed data. L.S. and D.C. provided technical support. J.X., L.S., Z.Q., L.W., and B.W. designed the research and interpreted data. J.X. wrote the manuscript. B.W. supervised the study, edited the manuscript and approved the submission.

## Supporting information

Supporting Information

Supporting Table 1

Supporting Table 2

Supporting Table 3

Supporting Table 4

## Data Availability

The data that support the findings of this study are available in the supplementary material of this article.
