# Supplementary material for: A Ubiquitin‐Dependent Switch on MEF2D Senses Pro‐Metastatic Niche Signals to Facilitate Intrahepatic Metastasis of Liver Cancer
Source: Adv Sci (Weinh). 2023 Oct 12;10(35):2305550. doi: 10.1002/advs.202305550 (PMC10724427; doi:10.1002/advs.202305550)
Supplement: Supplementary file 1 — Supporting Information [file ADVS-10-2305550-s004.pdf]

ADVANCED SCIENCE
Open Access

Supporting Information

for *Adv. Sci.*, DOI 10.1002/advs.202305550

A Ubiquitin-Dependent Switch on MEF2D Senses Pro-Metastatic Niche Signals to Facilitate Intrahepatic Metastasis of Liver Cancer

Junyu Xiang, Ni Zhang, Aibei Du, Jinyang Li, Mengyun Luo, Yuzhu Wang, Meng Liu, Luming Yang, Xianfeng Li, Lin Wang, Qin Liu, Dongfeng Chen, Tao Wang, Xiu-wu Bian, Zhong-yi Qin, Li Su*, Liangzhi Wen* and Bin Wang**

Supporting Information

A ubiquitin-dependent switch on MEF2D senses pro-metastatic niche signals to facilitate intrahepatic metastasis of liver cancer

Junyu Xiang, Ni Zhang, Aibei Du, Jinyang Li, Mengyun Luo, Yuzhu Wang, Meng Liu, Luming Yang, Xianfeng Li, Lin Wang, Qin Liu, Dongfeng Chen, Tao Wang, Xiu-wu Bian, Zhongyi Qin, Li Su, Liangzhi Wen, Bin Wang

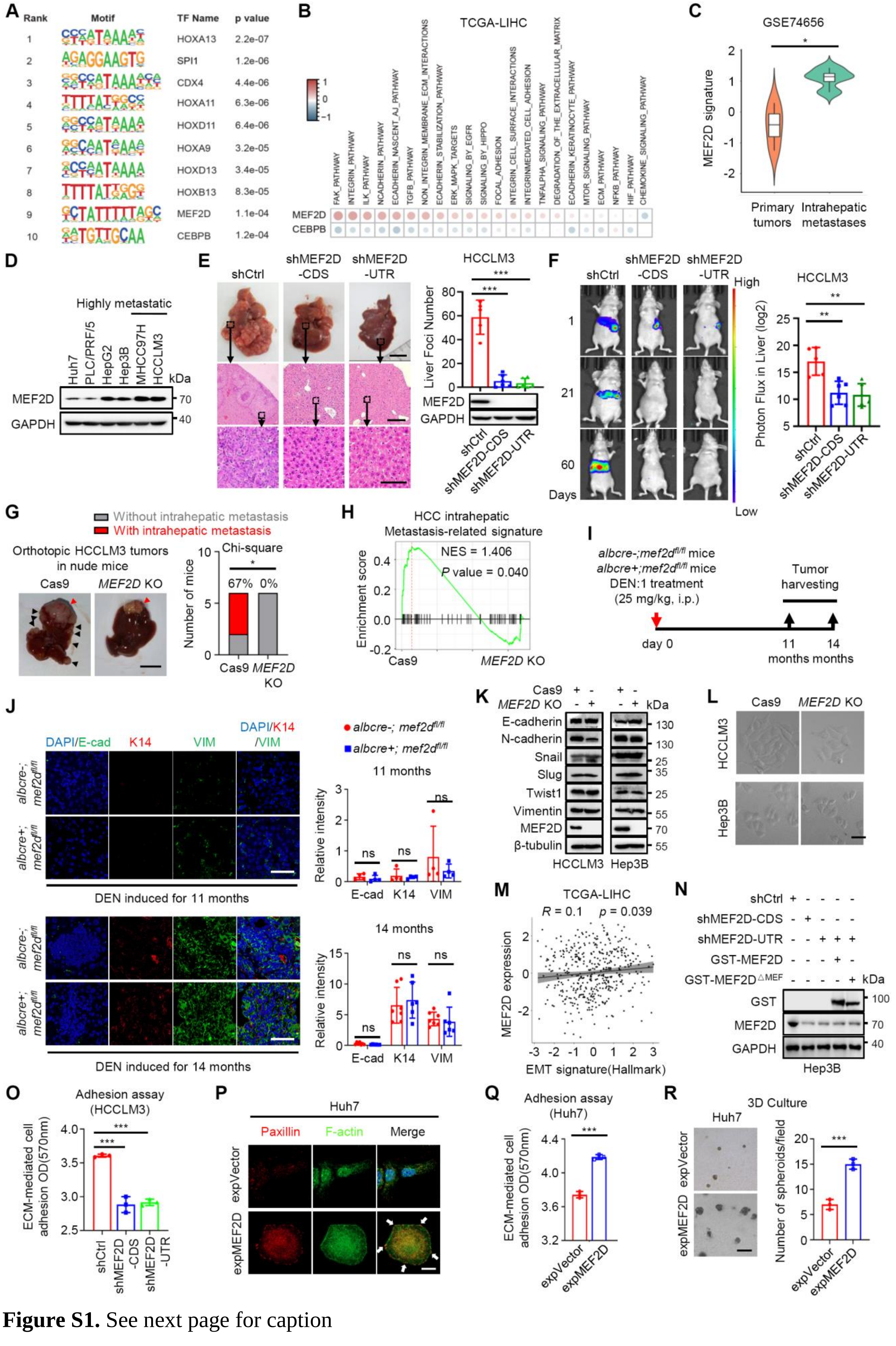

Figure S1. See next page for caption

Figure S1. The pro-metastatic niche signal-responsive transcription factor MEF2D promotes intrahepatic seeding of disseminated HCC cells independent of the EMT. A) The top 10 enriched transcription factor (TF) motifs shown in Figure 1A. B) Correlation analysis of MEF2D and CEBPB with signature genes among various pro-metastatic niche signaling pathways, using data from the TCGA-LIHC project. C) MEF2D signature of HCC[12] within primary HCC tissues and intrahepatic metastases of HCC from patients (n=4) (GSE74656). D) Immunoblot analysis of endogenous MEF2D expression in human HCC cell lines. The indicated cell lines have high metastatic potential. E) Livers of the *Mef2d*-depleted and control groups resected from a spleen-to-liver metastasis mouse model. The numbers of liver metastases were counted. Tissues were photographed, fixed, and stained with haematoxylin and eosin (H&E). Immunoblot to confirm the knockdown efficiency of *Mef2d* using shRNAs was shown. sh*Mef2d*-CDS or sh*Mef2d*-UTR, shRNAs targeting CDS or UTR of *Mef2d* mRNA respectively (n=5-6). Scale bar, 1 cm (top), 500 μm (middle), 100 μm (bottom). **F)** Bioluminescence imaging (BLI) at 1, 21, and 60 days after splenic injection of luciferase-labeled sh*Mef2d* or control HCCLM3 cells. The dots represent the photon flux of the BLI signal within a region that corresponds to the liver or spleen of each mouse (n=5-6). G) Intrahepatic metastases generated by the orthotopically inoculated *Mef2d*-depleted and control HCCLM3 cells in nude mice livers (n=6). Red arrow indicates primary tumor, while black arrow indicates metastases. Scale bars, 1 cm. H) GSEA plot analysis suggested that the enrichment of intrahepatic metastasis-related gene signature of HCC was decreased in *Mef2d*-depleted and control Hep3B cells. NES, normalized enrichment score. I) Schematic guideline for developing a DEN-induced mouse HCC model. J) Immunostaining for E-cadherin (E-cad), vimentin (VIM) and KRT14 (K14) in primary HCCs from DEN-treated $Alb\text{-}cre^{-};Mef2d^{fl/fl}$ control and $Alb\text{-}cre^{+};Mef2d^{fl/fl}$ mice (n=4-7). Scale bars, 50 μm. K-L) Immunoblot analysis of expression of EMT markers (K) and cellular morphology (L) of *Mef2d*-depleted and control HCCLM3 or Hep3B cells. Scale bar, 50 μm. Correlation analysis of *Mef2d* mRNA levels versus with the EMT gene signature using TCGA-LIHC datasets. The total least-squares regression line, confidence intervals, Pearson correlation coefficient (R) value and *P* value are shown. N) Immunoblot analysis of MEF2D in *Mef2d*-depleted Hep3B cells that were introduced with full-length MEF2D or MEF2D lacking the MEF domain. O) Crystal violet staining-based quantification of adhesion of indicated HCCLM3 cells to ECM. P) Redistribution of focal adhesions and cytoskeletal remodelling of control or MEF2D-overexpressing Huh7 cells on the ECM. The paxillin (red) and F-actin (green) was visualized by double immunostaining. White arrows indicate focal adhesions. Scale bar, 20 μm. Q) Crystal violet staining-based quantification of adhesion of control or MEF2D-overexpressing Huh7 cells to ECM. R) 3D growth of control or MEF2D-overexpressing Huh7 cells in ECM. Scale bar, 200 μm. All immunoblots are representative experiments of three independent replicates. For all panels, *** $P < 0.001$, and ns, no significance.

A

TCGA-LIHC

Gene	Expression	Recurrence		P value
		No	Yes	
MEF2A	Low	181	148	0.467
	High	17	18	
MEF2B	Low	186	143	0.012
	High	12	23	
MEF2C	Low	120	92	0.318
	High	78	74	
MEF2D	low	159	116	0.021
	high	39	50	

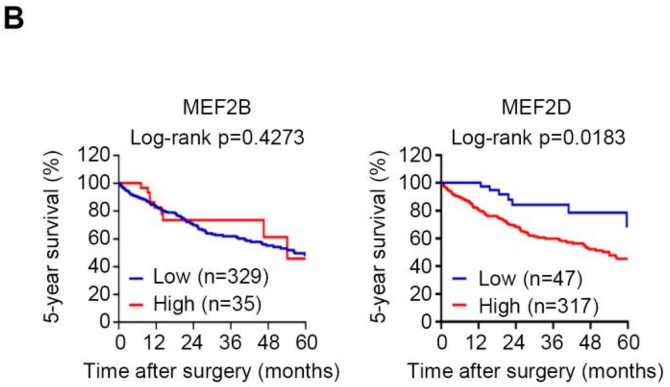

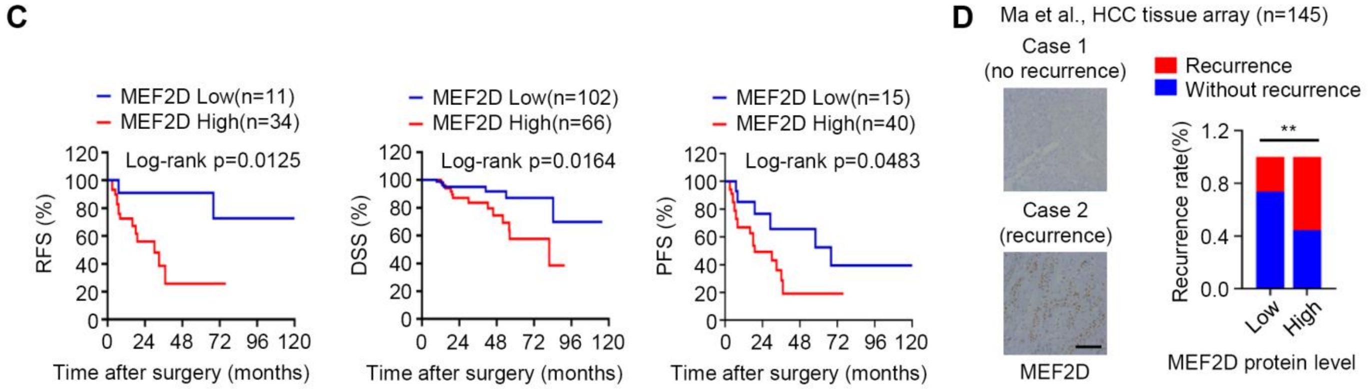

Figure S2. Elevated expression of MEF2D in tumor tissues correlates with post-surgery recurrence and survival of HCC patients. A) Correlation analysis of the expression levels of MEF2 family members and tumor relapse of surgery using a TCGA HCC dataset. B) Kaplan-Meier analysis of OS based on MEF2B or MEF2D expression in HCCs from TCGA database. C) Kaplan-Meier analysis of the relapse-free survival (RFS), disease-specific survival (DSS) and progression-free survival (PFS) of grade one HCC patients stratified by MEF2D expression using TCGA HCC datasets. D) Immunohistochemical analysis of MEF2D expression in HCC cells and its association with post-surgery recurrence (Cohort I). Representative IHC images of MEF2D staining using samples with or without disease recurrence. Scale bar,100 µm. ** $P < 0.01$.

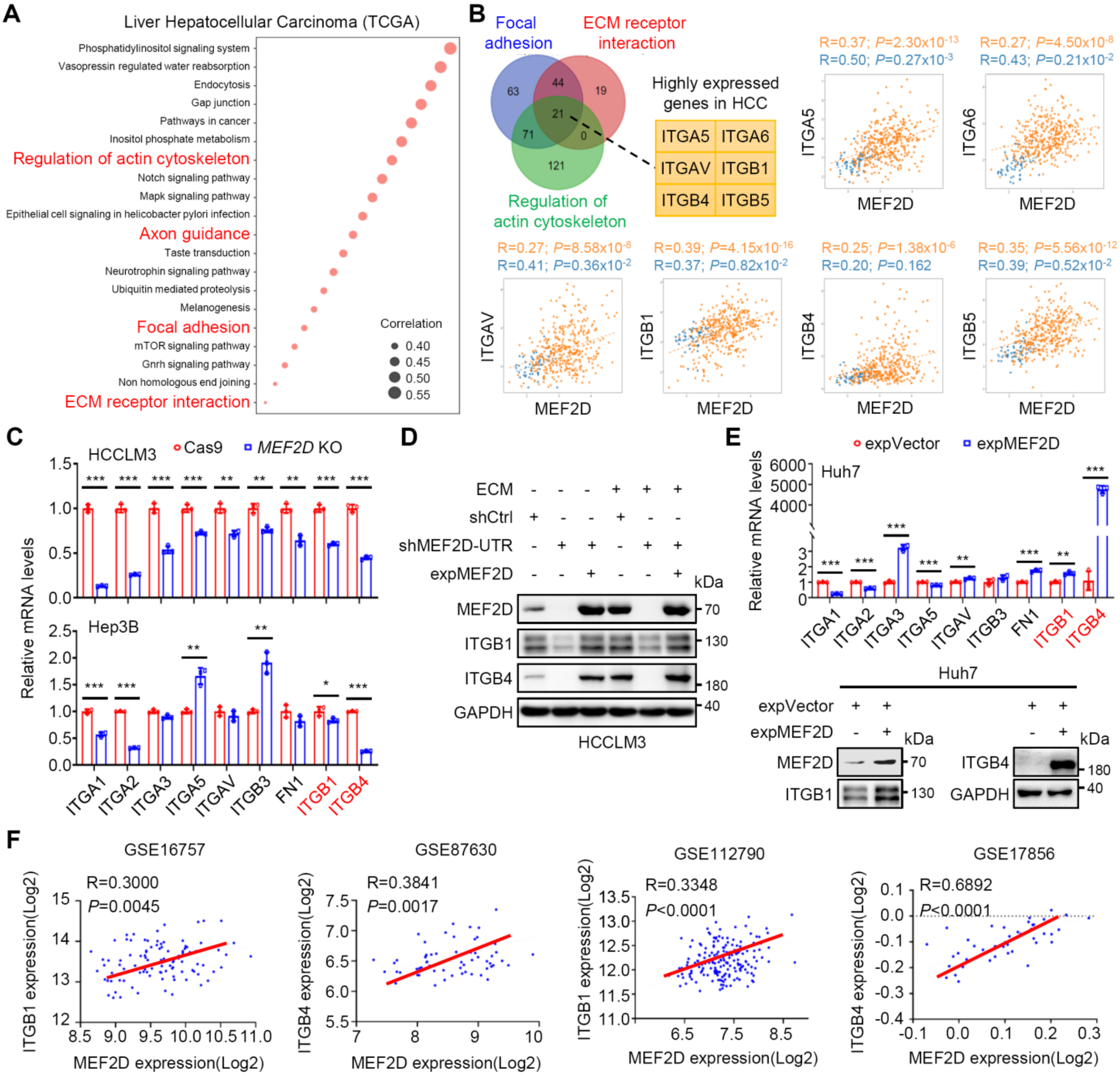

G

MEF2D & ITGB1 mRNA expression analysis	Log2 Odds Ratio	p-Value	q-Value	Tendency
Colorectal Adenocarcinoma (TCGA, PanCancer Atlas)	2.901	<0.001	<0.001	Co-occurrence
Kidney Renal Papillary Cell Carcinoma (TCGA, PanCancer Atlas)	>3	<0.001	<0.001	Co-occurrence
Ovarian Serous Cystadenocarcinoma (TCGA, PanCancer Atlas)	2.628	<0.001	<0.001	Co-occurrence
Cervical Squamous Cell Carcinoma (TCGA, PanCancer Atlas)	2.156	0.005	0.005	Co-occurrence
Glioblastoma Multiforme (TCGA, PanCancer Atlas)	>3	0.016	0.016	Co-occurrence
Bladder Urothelial Carcinoma (TCGA, PanCancer Atlas)	2.435	0.033	0.033	Co-occurrence
Breast Invasive Carcinoma (TCGA, PanCancer Atlas)	<-3	<0.001	<0.001	Mutual exclusivity

MEF2D & ITGB4 mRNA expression analysis	Log2 Odds Ratio	p-Value	q-Value	Tendency
Colorectal Adenocarcinoma (TCGA, PanCancer Atlas)	>3	<0.001	<0.001	Co-occurrence
Kidney Renal Papillary Cell Carcinoma (TCGA, PanCancer Atlas)	>3	0.004	0.004	Co-occurrence
Breast Invasive Carcinoma (TCGA, PanCancer Atlas)	-1.608	0.03	0.03	Mutual exclusivity

Figure S3. See next page for caption

Figure S3. MEF2D expression is positively associated with levels of *Itgb1* and *Itgb4* transcripts in human cancers. A) The top 20 MEF2D-related signalling pathways identified by analysing TCGA HCC datasets. B) Venn diagram analysis showing MEF2D-related signalling pathways and genes that were associated with focal adhesion, actin cytoskeleton dynamics, and ECM-receptor interaction, and also highly expressed in HCC. For the dot plots, yellow dots indicate gene expression in HCC tissues and blue dots indicate gene expression in adjacent normal tissue. C) qRT-PCR analysis of DEGs expression in *Mef2D*-depleted and control HCCLM3 or Hep3B cells. D) Immunoblot analysis of ITGB1 and ITGB4 levels in *Mef2D*-depleted and control HCCLM3 cells, or in *Mef2D*-depleted cells reconstituted with MEF2D. The culture plates were coated with or without ECM. E) qRT-PCR (upper) and immunoblot (lower) analysis of indicated integrin expression in MEF2D-overexpressing and control Huh7 cells. F) Correlation analysis of *Mef2D* and *Itgb1*/*Itgb4* mRNA levels using datasets from the Gene Expression Omnibus (GEO) HCC cohorts. G) Significant co-occurrence or mutual exclusivity of *Mef2D* and *Itgb1*/ *Itgb4* expression was observed in multiple cancer types. mRNA expression z-scores of RNA Seq V2 RSEM from available TCGA datasets were analyzed for significance, log odds ratio, and types of association. All immunoblots are representative experiments of three independent replicates. For all panels, * $P < 0.05$, ** $P < 0.01$, *** $P < 0.001$.

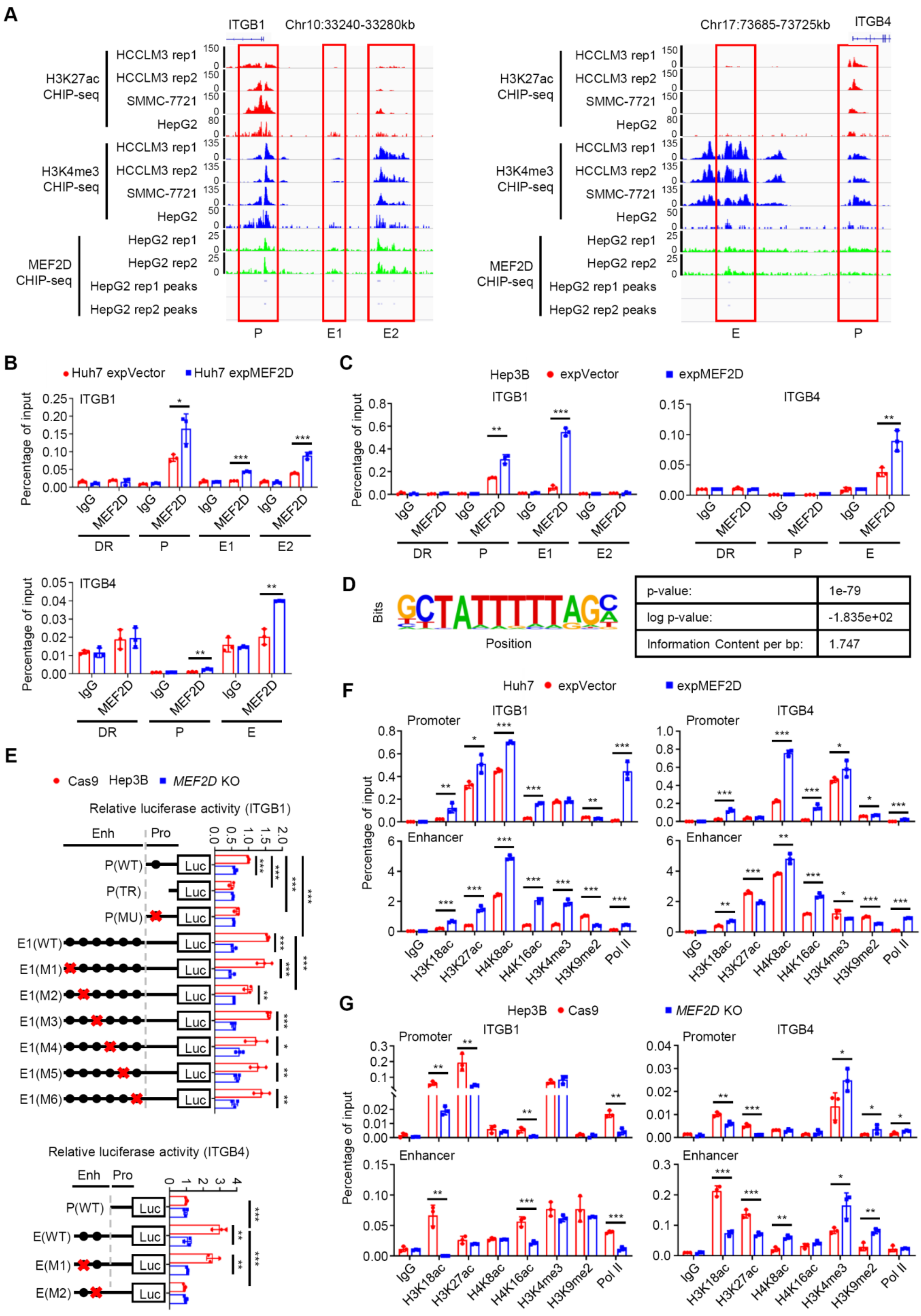

p-value:	1e-79
log p-value:	-1.835e+02
Information Content per bp:	1.747

Figure S4. See next page for caption

Figure S4. MEF2D transactivates *Itgb1* and *Itgb4* genes via binding their promoter and enhancer regions. A) Chromatin immunoprecipitation (ChIP) sequencing using MEF2D, H3K27ac and H3K4me3 antibodies demonstrated MEF2D binding to the promoter and enhancers of *Itgb1* and *Itgb4* genes in different HCC cell lines. P, promoter; E, enhancer. B-C) ChIP-qPCR analysis of MEF2D binding to the promoter and enhancer regions of *Itgb1* and *Itgb4* in Huh7 cells (B) or Hep3B cells (C). DR, distant region. D) Cistrome toolkit analysis of MEF2D ChIP-seq data in (A) identified the DNA-binding motif of MEF2D. E) Luciferase activities of *Itgb1* and *Itgb4* promoters were detected in *Mef2d*-depleted or control cells transfected with or without enhancer reporter constructs. F-G) ChIP analysis of Pol II, H3K18ac, H3K27ac, H4K8ac, H4K16ac, H3K4me3, H3K9me2 occupancy in the promoter and enhancer regions of *Itgb1* and *Itgb4* in MEF2D-expressing or control Huh7 cells (F) or in *Mef2d*-depleted or control Hep3B cells (G). An isotype-matched IgG was used as a negative control. For all panels, * $P < 0.05$, ** $P < 0.01$, *** $P < 0.001$.

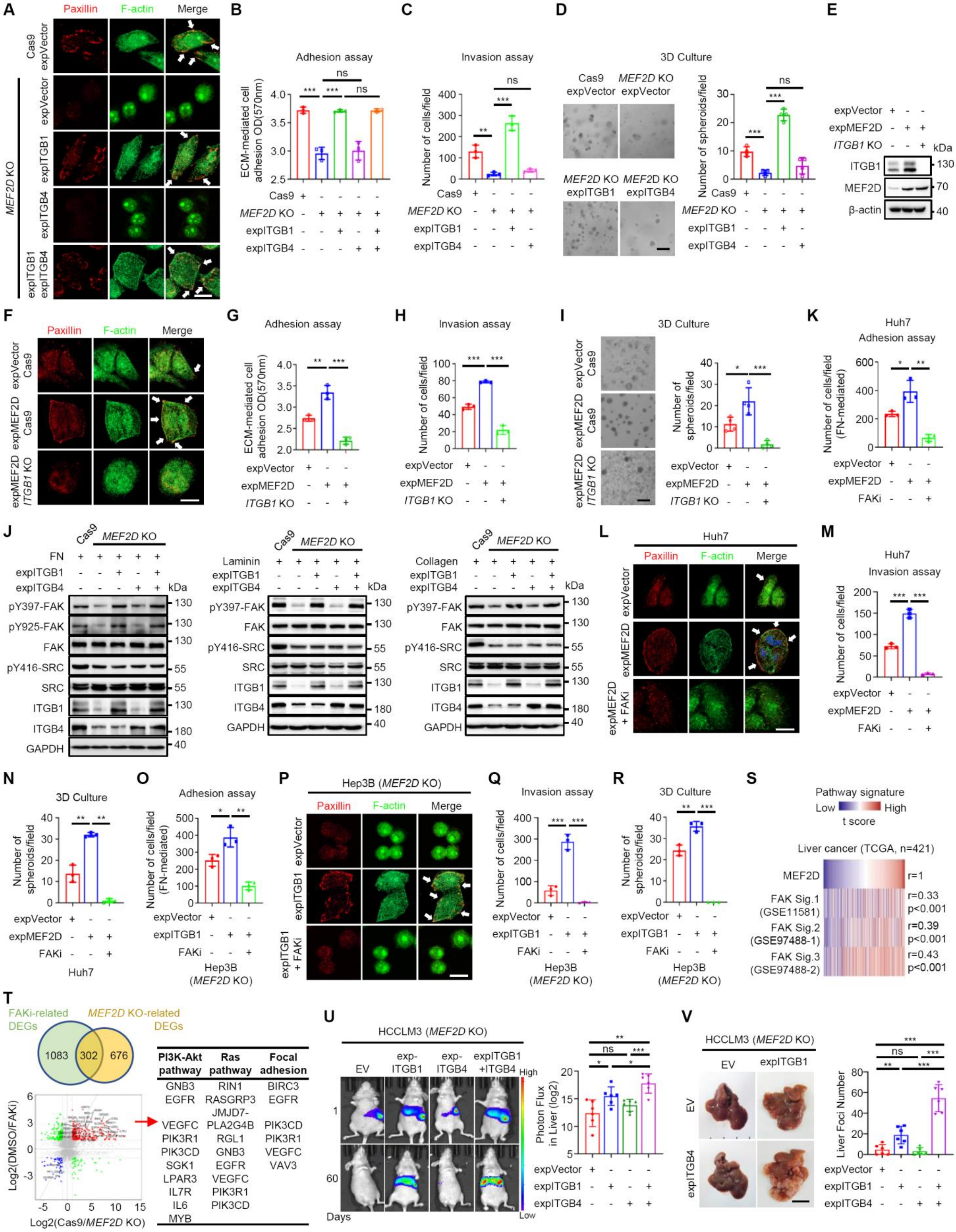

Figure S5. See next page for caption

Figure S5. ITGB1, but not ITGB4, plays an important role in MEF2D-driven seeding of disseminated HCC cells on the ECM via activating FAK signalling pathway. A) Redistribution of focal adhesions and cytoskeletal remodelling in *Mef2d*-depleted Hep3B cells that were reconstituted with either *Itgb1*, or *Itgb4*, or both, then cultured on the ECM for double immunostaining. Paxillin, red. F-actin, green. White arrows indicate focal adhesions. Scale bar, 20 μm. B) Crystal violet staining-based quantification of the adhered Hep3B cells in ECM. C) Quantitation of invaded Hep3B cells in an ECM-coated transwell assay. D) Anchorage-independent growth of the indicated Hep3B cells in ECM. Scale bar, 200 μm. E) Immunoblot analysis of the knockdown efficiency of *Itgb1* in MEF2D-overexpressing Huh7 cells. F) Co-immunostaining to visualize the redistribution of focal adhesions and cytoskeletal remodelling in Huh7 cells on the ECM. White arrows indicate focal adhesions. Scale bar, 20 μm. G-H) Crystal violet staining to quantify cell adhesion (G) and invasiveness (H) of Huh7 cells on ECM. I) Anchorage-independent growth of Huh7 cells in ECM. Scale bar, 200 μm. J) *Mef2d*-depleted Hep3B cells were reconstituted with *Itgb1*, or *Itgb4*, or both, and then cultured on fibronectin (FN), laminin (bottom coated, 10 μg/ml, left), or collagen (bottom coated, 10 μg/ml, right). Immunoblotting was performed to analyze FAK and SRC signaling activity. K) Quantification of the adhesion capacity of control or MEF2D-expressing Huh7 cells to FN, or cells that were pre-treated with the FAK inhibitor PF562271 (10 μM). L) Redistribution of focal adhesions and cytoskeletal remodelling in the indicated Huh7 cells on the ECM were examined by double immunostaining for paxillin (red) and F-actin (green). White arrows indicate focal adhesions. Scale bar, 20 μm. M-N) Quantitation of the invasiveness after 48 hours (M) and anchorage-independent growth after 5 days (N) of indicated Huh7 cell in ECM. O-P) *Mef2d*-depleted Hep3B cells were reconstituted with *Itgb1*, with or without pre-treatment with the FAK inhibitor PF562271 (10 μM). Cell adherence to FN was quantified in (O). Redistribution of focal adhesions and cytoskeletal remodelling of cells on ECM (P) were examined by double immunostaining for paxillin (red) and F-actin (green). White arrows indicate focal adhesions. Scale bar, 20 μm. Q-R) Cell invasiveness after 48 hours (Q) and 3D growth after 5 days (R) in ECM were measured. S) Pearson's correlations between the *Mef2d* mRNA level and FAK signatures were analyzed using different GEO databases (GSE11581 and GSE97488). T) Venn diagram of DEGs by analysing the transcriptome of *Mef2d*-depleted or FAK inhibited HCC cells. Downregulated pathways and genes in both *Mef2d*-depleted vs Ctrl HCC cells[14] and FAK inhibited HCC cells vs Ctrl HCC cells. (U) BLI at 1 and 60 days after splenic injection of *Mef2d*-depleted HCCLM3 cells that were reconstituted with either *Itgb1*, or *Itgb4*, or both (n=5-6). V) Liver metastases of the indicated HCCLM3 cells after splenic injection were counted. Scale bar, 1 cm. All immunoblots are representative experiments of three independent replicates. For all panels, * $P < 0.05$, ** $P < 0.01$, *** $P < 0.001$, ns, no significance.

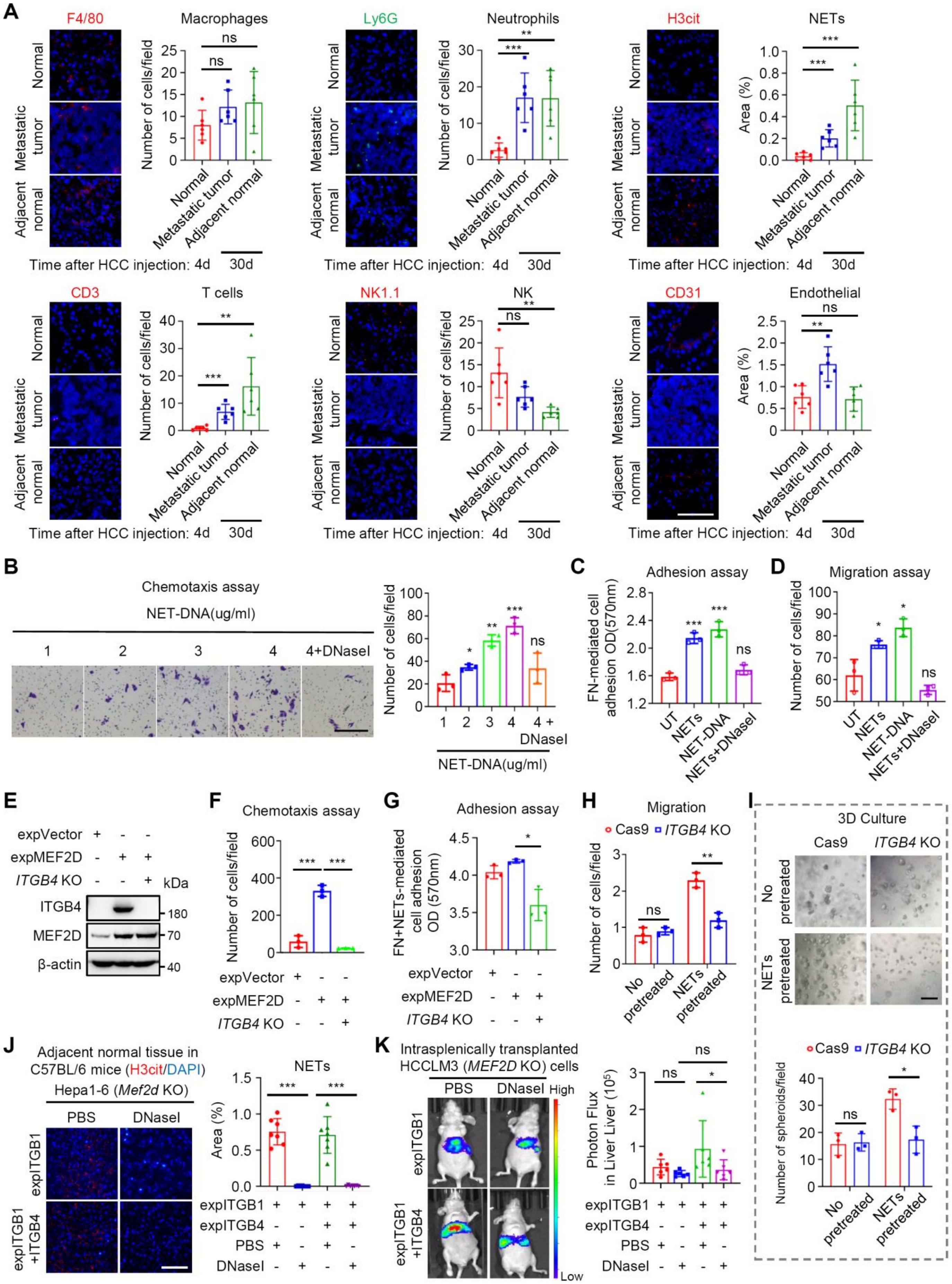

Figure S6. See next page for caption

Figure S6. NETs enriched in later colonization stage of the pro-metastatic niche attract DCCs via interacting with ITGB4, another transcriptional target of MEF2D. A) Representative immunofluorescence images staining macrophages, neutrophils, NETs, T cells, NK cells, and endothelial cells in normal liver tissues of C57BL/6 mice 4 days after intrahepatic injection of Hepa1-6 cells, or in the metastatic tumors or adjacent normal tissues 30 days after Hepa1-6 cell injection. Scale bars, 100 μm. The quantitation results are shown. B) Chemotaxis of disseminated cells in response to NETs-DNA. Hep3B cells were plated in the upper chambers and NETs-DNA were added to culture media in increasing concentrations (0-5 μg/ml), with or without DNase I pre-treatment in the lower chambers of transwell assay. Scale bars, 100 μm. C-D) Cell adhesion (C) and migration (D) induced by NETs-DNA. Hep3B cells were stimulated with 5 μg/ml NET-DNA or 5 μg/ml NETs in the presence or the absence of DNase I. E) Immunoblot analysis of *Itgb4* knockdown efficiency in MEF2D-overexpressing Huh7 cells. Immunoblots are representative of three independent replicates. F) Chemotaxis of control or MEF2D-overexpressing, or MEF2D-overexpressing and *Itgb4*-depleted cells induced by NETs-DNA. Huh7 were seeded in the upper chambers and NK cells or NETs were added to the culture media in the lower chambers of transwell assay. Tumor cells:NK cells/NETs = 1:5. G) Crystal violet staining to quantify the indicated Huh7 cell adhesion induced by NETs-DNA (5 μg/ml) and FN (10 μg/ml). H) Migration of *Itgb4*-depleted or control Hep3B cells with or without NETs pre-treatment. I) 3D growth of indicated Hep3B cells in ECM. Scale bar, 200 μm. J) Representative immunofluorescence images staining NETs in the adjacent normal tissues of metastatic tumors 30 days after intrahepatic injection of the Hepa1-6 cells followed by treated with or without DNase I (5 mg/kg) (n=7). Scale bars, 100 μm. The quantification results are shown. K) BLI of liver metastases of *Mef2d*-depleted HCCLM3 cells reconstituted with either *Itgb1*, or both *Itgb1* and *Itgb4*. Cells were injected into the spleens of mice which were subsequently treated with DNase I (5 mg/kg) (n=6). Scale bar, 1 cm. All immunoblots are representative experiments of three independent replicates. For all panels, * $P < 0.05$, ** $P < 0.01$, *** $P < 0.001$, and ns, no significance.

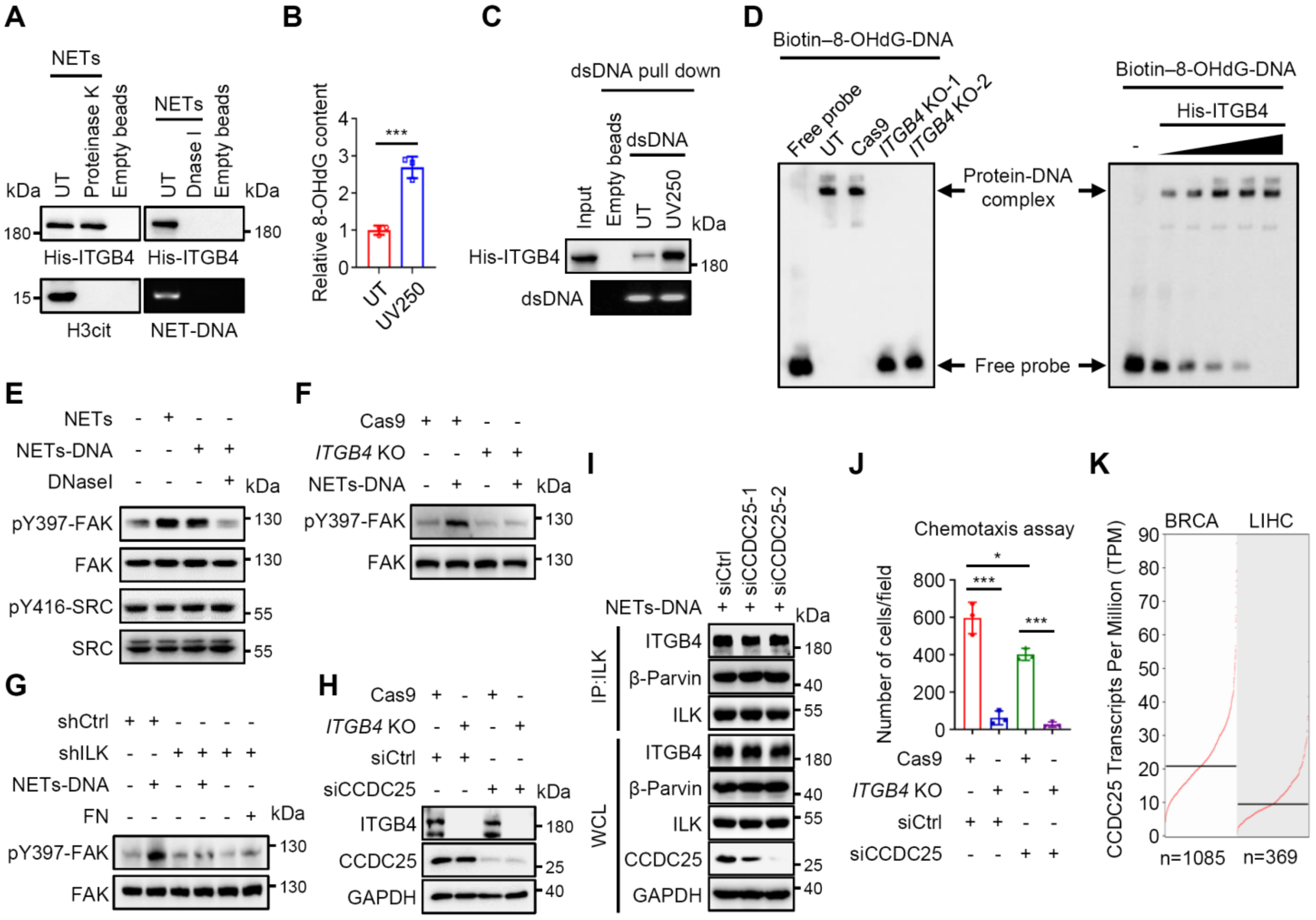

Figure S7. ITGB4 acts as a novel receptor for NETs-DNA to activate the prometastatic FAK signalling pathway in DCCs, a process that is independent of CCDC25. A) ITGB4 specifically interacts with NETs *in vitro*. Purified NETs were coupled to magnetic beads, treated with Proteinase K (left) or DNase I (right), and incubated with His-ITGB4. The interaction of NETs and ITGB4 was evaluated by the precipitation of NETs-beads and blotted with anti-His antibody. His-ITGB4 mixed with beads without DNA served as a negative control (empty beads). The digestion efficiency of the protein and DNA components of NETs by Proteinase K and DNase I was confirmed by immunoblotting for H3cit and agarose gel analysis for DNA, resepectively. B) Biotinylated heterologous 90-bp DNA duplexes with random sequences were either treated with or without UV-C light. The relative 8-OHdG content in the DNA was determined by ELISA. C) Pull-down assay revealed that His-ITGB4 preferentially interacts UV250-treated dsDNA. His-ITGB4 mixed with beads without DNA served as a negative control (empty beads). D) EMSA revealed the binding of biotinylated 8-OHdG-DNA to membrane proteins in control but not *ITGB4*-depleted cells (left), and specific interaction of biotinylated 8-OHdG-DNA with His-ITGB4 (right). The protein-DNA complex and free probe were denoted. E-G) Immunoblot analysis for FAK and SRC phosphorylation in Hep3B cells that were treated with NETs, NETs-DNA, or DNase I (E), in control or *ITGB4*-depleted cells that were treated with NETs-DNA (F), or in control or *ILK*-depleted cells that were treated with NETs-DNA or FN(G). H) Immunoblot analysis for ITGB4 and CCDC25 protein levels in Hep3B cells in control or *Itgb4* knockout and/or *CCDC25*-depleted cells. I) Immunoblot analysis of anti-ILK immunoprecipitates in control or *CCDC25*-depleted Hep3B cells, suggesting that in the presence of NETs-DNA, the interaction between ILK, ITGB4 and β-Parvin were not mediated by CCDC25. J) Chemotaxis of control, *Itgb4* knockout and/or *CCDC25*-depleted Hep3B cells induced by NETs-DNA. Hep3B cells were seeded in the upper chamber and NETs were added to the culture media in the lower chamber of transwell assay. Tumor cells:NETs = 1:5. K)

CCDC25 expression level in samples of breast carcinoma or hepatocellular carcinoma patients from TCGA and GTEx databases (gepia2.cancer-pku.cn/). BRCA, breast invasive carcinoma; LIHC, Liver hepatocellular carcinoma. All immunoprecipitation and immunoblots are representative experiments of three independent replicates. For all panels, * $P < 0.05$, ** $P < 0.01$, *** $P < 0.001$, and ns, no significance.

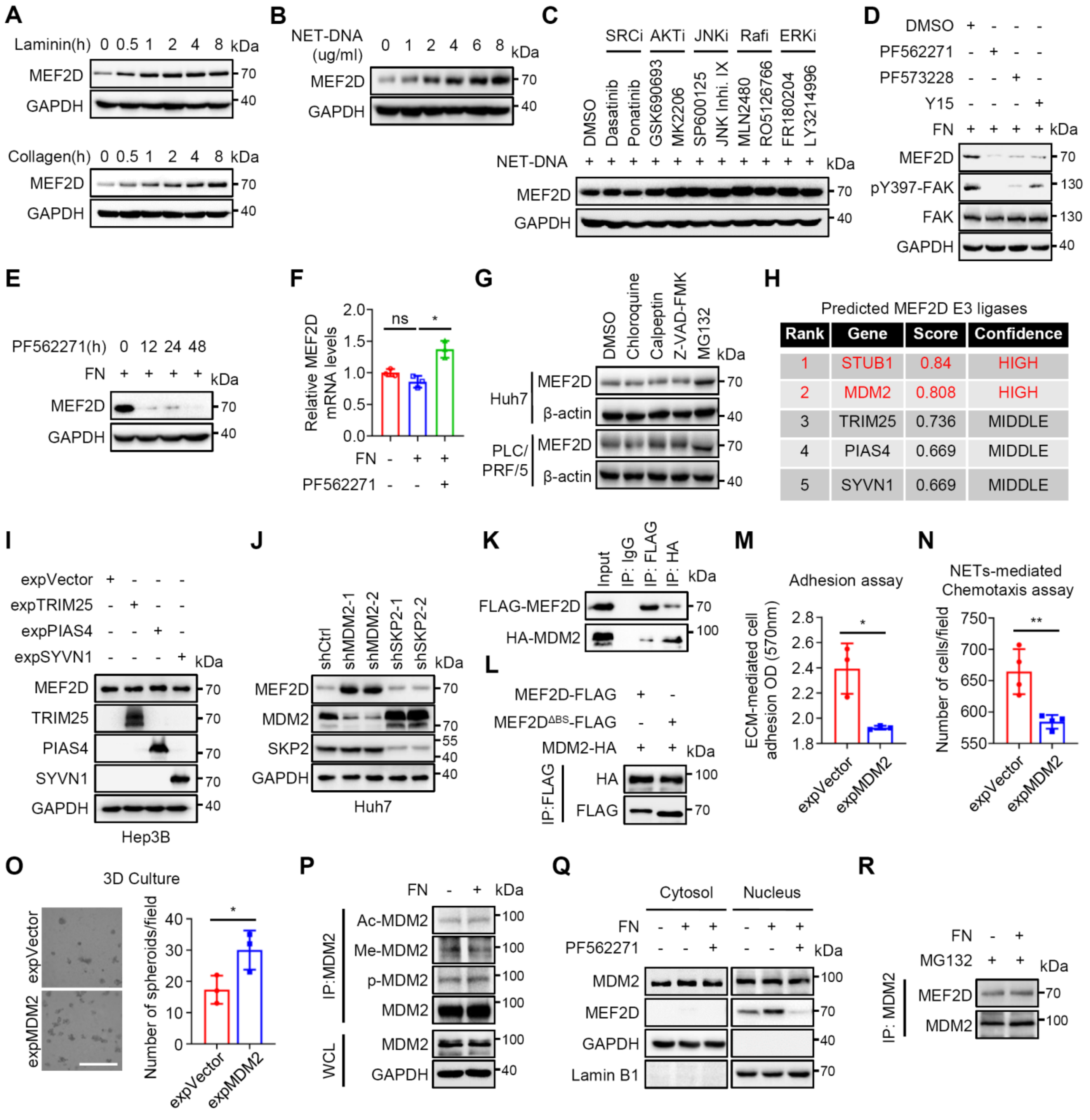

Figure S8. Pro-metastatic niche signals inhibit MDM2-mediated degradation of MEF2D *via* an integrin-FAK feedback loop. A-B) Immunoblot analysis of MEF2D protein levels in Hep3B cells treated with laminin or collagen (A), or NETs-DNA (B). C-D) Immunoblot analysis of Hep3B cells treated with NET-DNA together with inhibitors against SRC, AKT, JNK, RAF or ERK kinases respectively for 12 hours (C), or incubated with FN together with DMSO or 10 μM FAK inhibitors PF562271, PF573228, or Y15 (D). E-F) Analysis of MEF2D protein levels by immunoblot (E) and mRNA levels by qRT-PCR (F) in Hep3B cells cultured on FN, in the presence of FAK inhibitor PF562271 (10 μM). G) Immunoblot analysis of MEF2D protein levels in Huh7 and PLC/PRF/5 HCC cells treated with DMSO, or inhibitors of autophagy (chloroquine), calpain (calpeptin), pan-caspase (Z-VAD-FMK), or proteasome (MG132). H) Predicted MEF2D E3 ligases and their respective confidence level from UbiBrowser 2.0 (http://ubibrowser.ncpsb.org.cn/v2/). I) Immunoblot of MEF2D protein levels in Hep3B cells expressing

either TRIM25, PIAS4, or SYVN1. J) Immunoblot analysis of MEF2D protein levels in *MDM2*-depleted or *SKP2*-depleted Huh7 cells. K) Co-IP analysis of the interaction between exogenous Flag-MEF2D and HA-MDM2 in HEK293T cells. L) Co-IP analysis of the association between HA-MDM2 with exogenous Flag-MEF2D or Flag-MEF2D lacking the predicted binding motif in HEK293T cells. M) Crystal violet staining to quantify adhesion of control or MDM2-overexpressing Hep3B cells to ECM. N) Chemotaxis of control or MDM2-overexpressing Hep3B cells induced by NETs in the lower chamber of transwell. Tumor cells:NETs = 1:5. O) 3D growth of Hep3B cells in ECM. Scale bars, 10 μm. P) IP analysis of MDM2 acetylation, methylation, and phosphorylation in Hep3B cells treated with FN (bottom coated, 10 μg/ml). Q) Immunoblot analysis of MDM2 and MEF2D protein levels in cytosolic and nuclear fraction of Hep3B cells. Cells were treated with or without FN and FAK inhibitor PF562271 (10 μM). GAPDH and lamin B were used as quality controls for the cytosolic and nuclear fractions, respectively. R) Co-IP analysis of the association between endogenous MEF2D and MDM2 in Hep3B cells with FN treatment. All immunoprecipitation and immunoblot are representative experiments of three independent replicates. For all panels, * $P < 0.05$, ** $P < 0.01$, and ns, no significance.

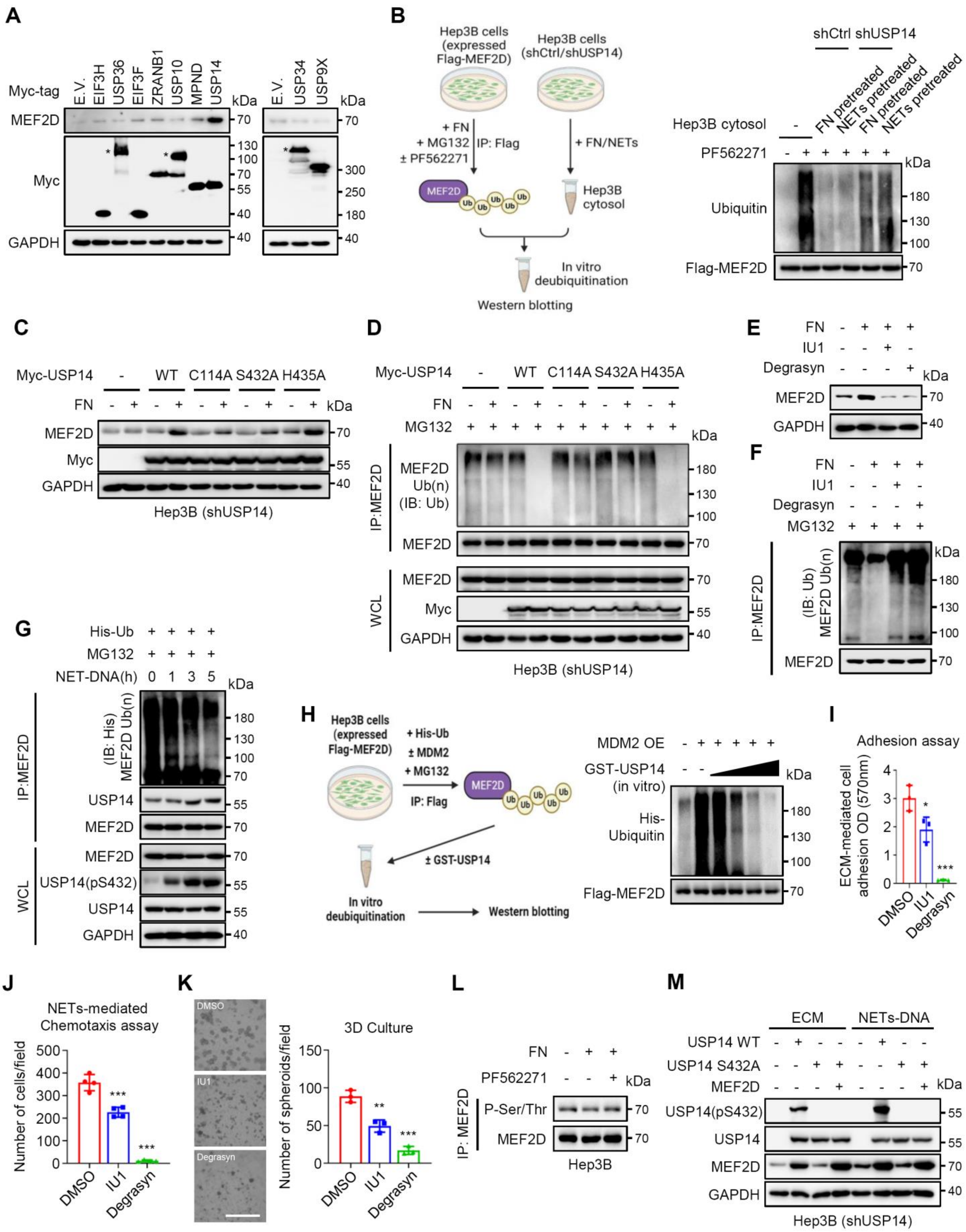

Figure S9. See next page for caption

Figure S9. USP14 is phosphorylated and recruited by the integrin-FAK signalling to stabilize MEF2D and amplify the pro-metastatic feedback loop. A) Immunoblot analysis of MEF2D protein levels in Huh7 cells transfected with Myc-tagged DUB constructs (related to Figure 5D). * marks the band corresponding to the indicated protein. E.V., empty vector. B) Schematic model of an *in vitro* deubiquitination assay (left). The ubiquitinated MEF2D protein was immunoprecipitated from Hep3B cells treated with FN (bottom coated, 10 μg/ml), MG132 (10 μM), and FAK inhibitor PF562271 (10 μM). The purified Flag-MEF2D protein was incubated with the cytosolic fraction of control and *USP14*-depleted Hep3B cells, in the presence of FN or NETs (5 μg/ml), and analyzed by immunoblot (right). C-D) *USP14*-depleted Hep3B cells were transfected with wild-type USP14 (WT) or USP14 mutant constructs (C114A, S432A and H435A), and incubated with or without FN. The MEF2D protein levels (C) and its polyubiquitination status (D) were examined by immunoblot of cell lysates and MEF2D immunoprecipitates. E-F) Hep3B cells were treated with or without FN and USP14 inhibitors IU1 (30 μM) or Degrasyn (10 μM) to examine the MEF2D protein levels (E) and its polyubiquitination status (F) by immunoblotting. G) MEF2D polyubiquitination, and its interaction with USP14, and USP14-pS432 levels in Hep3B cells exposed to NETs-DNA for the indicated times. H) Schematic model of an *in vitro* deubiquitination assay (left). The ubiquitinated MEF2D was immunoprecipitated from MG132 treated Hep3B cells expressing His-ubiquitin and HA-MDM2, then incubated with purified GST-USP14, and analyzed by immunoblot (right). I-K) Hep3B cells were treated with or without USP14 inhibitors IU1 (30 μM) or Degrasyn (10 μM). Crystal violet staining were performed to quantify of adhesion of disseminated cells to ECM (I). Chemotaxis of the cells induced by NETs were examined in a transwell assay (J), Tumor cells:NETs = 1:5. 3D growth of cells were examined in ECM (K). Scale bars, 10 μm. L) Immunoblot analysis of pSer/pThr on purified MEF2D in Hep3B cells treated with or without FN and PF562271. M) *USP14*-depleted Hep3B cells were transfected with USP14 WT or USP14 S432A mutant or together with MEF2D constructs, and co-cultured with ECM or NETs-DNA. USP14-pS432 levels and MEF2D protein expression were assessed by immunoblotting. All immunoprecipitation and immunoblot are representative experiments of three independent replicates. For all panels, * $P < 0.05$, ** $P < 0.01$, *** $P < 0.001$.

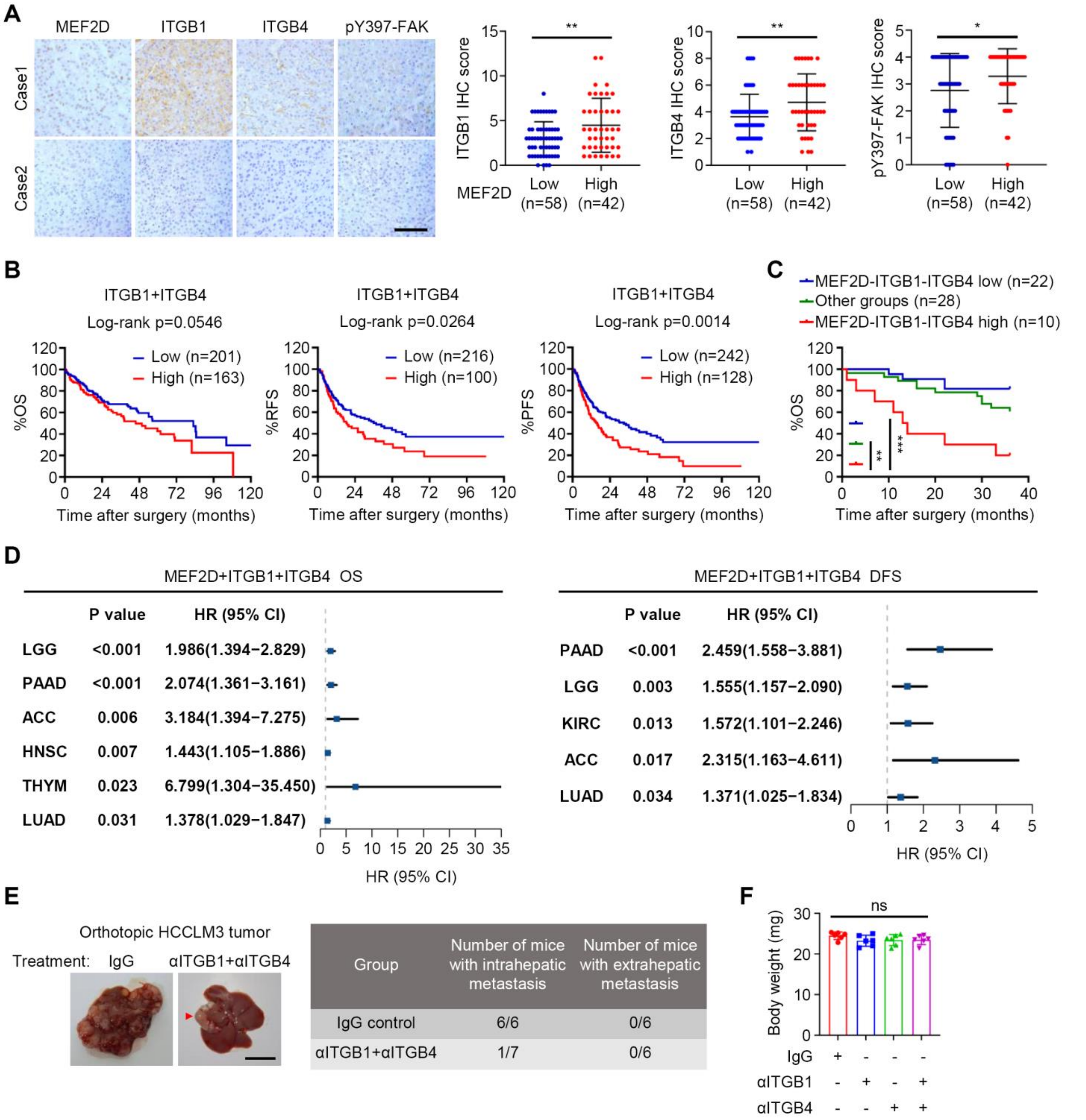

Figure S10. Elevated expression of MEF2D-ITGB1/ITGB4 axis predicts shorter relapse-free survival in multiple human cancers, while blockade of both ITGB1 and ITGB4 significantly inhibits intrahepatic metastasis of HCC cells. A) Immunohistochemical (IHC) analysis and correlation analysis of MEF2D, ITGB1, ITGB4, and pY397-FAK levels in tumour tissues from 100 HCC patients (Cohort III). Shown are representative IHC images from two samples, revealing their positive correlations. Scale bar: 100 μm. B) According to the co-expression status of ITGB1 and ITGB4 in tumour tissues using TCGA dataset, HCC patients were stratified for Kaplan-Meier analysis of overall survival (OS), relapse-free survival (RFS) and progression-free survival (PFS). C) Kaplan-Meier analysis of 3-year overall survival of 60 patients with HCC (Cohort III) stratified by the expression status of MEF2D, ITGB1 and ITGB4 in their tumor tissues. D) Association analysis between OS or DFS and the expression status of MEF2D, ITGB1 and ITGB4 in cancer

tissues, using indicated cancer sequencing data from TCGA database. LGG, Brain Lower Grade Glioma; PAAD, Pancreatic adenocarcinoma; ACC, Adrenocortical carcinoma; HNSC, Head and Neck squamous cell carcinoma; THYM, Thymoma; LUAD, Lung adenocarcinoma; KIRC, Kidney renal clear cell carcinoma. E) Livers of nude mice orthotopically injected with HCCLM3 cells to develop intrahepatic metastasis. Mice were treated by both anti-ITGB1 and anti-ITGB4 antibodies, with IgG served as a negative control. Metastases were analyzed in the livers and other organs. Red arrow indicated the primary tumors. Scale bars, 1 cm. F) Body weight of the mice from experiments shown in Figure 7D-F.
